# Premastectomy Radiotherapy and Immediate Breast Reconstruction: A Randomized Clinical Trial

**DOI:** 10.1001/jamanetworkopen.2024.5217

**Published:** 2024-04-05

**Authors:** Mark V. Schaverien, Puneet Singh, Benjamin D. Smith, Wei Qiao, Catherine L. Akay, Elizabeth S. Bloom, Mariana Chavez-MacGregor, Carrie K. Chu, Mark W. Clemens, Jessica S. Colen, Richard A. Ehlers, Rosa F. Hwang, Melissa M. Joyner, Rene D. Largo, Alexander F. Mericli, Melissa P. Mitchell, John W. Shuck, Nina Tamirisa, Debasish Tripathy, Mark T. Villa, Wendy A. Woodward, Rensi Zacharia, Henry M. Kuerer, Karen E. Hoffman

**Affiliations:** 1 Division of Surgery, Department of Plastic Surgery, The University of Texas MD Anderson Cancer Center, Houston; 2 Division of Surgery, Department of Breast Surgical Oncology, The University of Texas MD Anderson Cancer Center, Houston; 3 Division of Radiation Oncology, Department of Breast Radiation Oncology, The University of Texas MD Anderson Cancer Center, Houston; 4 Department of Biostatistics, The University of Texas MD Anderson Cancer Center, Houston; 5 Division of Cancer Medicine, Department of Breast Medical Oncology, The University of Texas MD Anderson Cancer Center, Houston

## Abstract

**Importance:**

Premastectomy radiotherapy (PreMRT) is a new treatment sequence to avoid the adverse effects of radiotherapy on the final breast reconstruction while achieving the benefits of immediate breast reconstruction (IMBR).

**Objective:**

To evaluate outcomes among patients who received PreMRT and regional nodal irradiation (RNI) followed by mastectomy and IMBR.

**Design, Setting, and Participants:**

This was a phase 2 single-center randomized clinical trial conducted between August 3, 2018, and August 2, 2022, evaluating the feasibility and safety of PreMRT and RNI (including internal mammary lymph nodes). Patients with cT0-T3, N0-N3b breast cancer and a recommendation for radiotherapy were eligible.

**Intervention:**

This trial evaluated outcomes after PreMRT followed by mastectomy and IMBR. Patients were randomized to receive either hypofractionated (40.05 Gy/15 fractions) or conventionally fractionated (50 Gy/25 fractions) RNI.

**Main Outcome and Measures:**

The primary outcome was reconstructive failure, defined as complete autologous flap loss. Demographic, treatment, and outcomes data were collected, and associations between multiple variables and outcomes were evaluated. Analysis was performed on an intent-to-treat basis.

**Results:**

Fifty patients were enrolled. Among 49 evaluable patients, the median age was 48 years (range, 31-72 years), and 46 patients (94%) received neoadjuvant systemic therapy. Twenty-five patients received 50 Gy in 25 fractions to the breast and 45 Gy in 25 fractions to regional nodes, and 24 patients received 40.05 Gy in 15 fractions to the breast and 37.5 Gy in 15 fractions to regional nodes, including internal mammary lymph nodes. Forty-eight patients underwent mastectomy with IMBR, at a median of 23 days (IQR, 20-28.5 days) after radiotherapy. Forty-one patients had microvascular autologous flap reconstruction, 5 underwent latissimus dorsi pedicled flap reconstruction, and 2 had tissue expander placement. There were no complete autologous flap losses, and 1 patient underwent tissue expander explantation. Eight of 48 patients (17%) had mastectomy skin flap necrosis of the treated breast, of whom 1 underwent reoperation. During follow-up (median, 29.7 months [range, 10.1-65.2 months]), there were no locoregional recurrences or distant metastasis.

**Conclusions and Relevance:**

This randomized clinical trial found PreMRT and RNI followed by mastectomy and microvascular autologous flap IMBR to be feasible and safe. Based on these results, a larger randomized clinical trial of hypofractionated vs conventionally fractionated PreMRT has been started (NCT05774678).

**Trial Registration:**

ClinicalTrials.gov Identifier: NCT02912312

## Key Points

**Question:**

Is premastectomy radiotherapy (PreMRT), followed by mastectomy and immediate breast reconstruction (IMBR), feasible and safe?

**Findings:**

In this randomized clinical trial with 49 patients, patients received either hypofractionated (40.05 Gy/15 fractions; n = 24) or conventionally fractionated (50 Gy/25 fractions; n = 25) regional nodal irradiation including the internal mammary chain. Mastectomy with IMBR was performed at a median of 23 days after radiotherapy; there were no complete flap losses and no recurrences at a median of 29.7 months of follow-up.

**Meaning:**

This study found that PreMRT followed by IMBR with autologous microvascular flap breast reconstruction is feasible and safe, shortening the time to complete breast reconstruction.

## Teaser

This randomized clinical trial evaluates outcomes among patients who received premastectomy radiotherapy and regional nodal irradiation followed by mastectomy and immediate breast reconstruction.

## Introduction

Among patients with breast cancer who require mastectomy and radiotherapy (RT) and desire breast reconstruction, the sequencing of RT and reconstruction remains a clinical challenge.^1,2,3,4,5,6,7^ At present, breast reconstruction algorithms for patients requiring postmastectomy RT (PMRT) focus predominantly on avoiding delivery of radiation to the definitive reconstruction to avoid late toxic effects and consequent negative effects on patient satisfaction.^1,2,3,4,5,6,7^ These strategies include staged reconstruction with placement of a temporary tissue expander (TE) at the time of mastectomy,^8^ which is associated with high rates of cellulitis and explantation,^9^ which can delay RT^10,11^; alternatively, patients may undergo mastectomy without reconstruction. Delayed reconstruction is typically performed approximately 6 to 12 months after PMRT.^9,12,13^ Immediate breast reconstruction (IMBR) at the time of mastectomy has numerous advantages compared with delayed reconstruction, including performance of mastectomy and reconstruction in a single operation, reduced treatment costs, superior cosmetic results, and improved psychosocial patient-reported outcomes.^13,14,15^

Preoperative RT is well established in the treatment of several types of cancer that are radiosensitive, such as esophageal carcinoma, rectal carcinoma, and sarcoma,^16,17^ and has also been combined with neoadjuvant systemic therapy to render unresectable, locally advanced breast cancers operable.^18,19,20^ Premastectomy RT (PreMRT) is a new sequence approach to RT delivery in breast cancer to facilitate IMBR. In this sequence, RT is delivered to the intact breast and regional lymphatics before surgery, allowing patients to undergo definitive IMBR at the time of mastectomy while avoiding the adverse effects of radiation on healthy donor tissues and the risk of delay of adjuvant RT.

The few studies that have evaluated the PreMRT treatment sequence indicate that it is feasible and safe, although most studies are historical and retrospective.^18,19,20,21,22,23,24^ However, the safety of microvascular reconstruction in the setting of regional nodal irradiation (RNI) including the internal mammary lymph nodes, which is the standard of care for RT for node-positive breast cancer in the US, has not yet been established.^25^ Although randomized clinical trials have demonstrated that shorter-course RT to the intact breast provides equivalent cancer control while reducing RT adverse effects. including lymphedema and skin or soft tissue toxic effects, compared with longer-course RT, it has not been well studied in the setting of RNI and is not yet considered standard of care by many radiation oncologists.^26,27^ In addition, the effect of dose fractionation on aesthetic, oncologic, and adverse outcomes in the preoperative setting is unresolved.

This study, to our knowledge, was the first of its type in the US, a prospective phase 2 randomized clinical trial of PreMRT for patients with breast cancer. Patients were randomized to receive either hypofractionated (HF) or conventionally fractionated (CF) RNI, followed by mastectomy and IMBR. The objective was to examine complication rates and outcomes to address knowledge gaps about the feasibility and safety of IMBR in the setting of RNI. The primary outcome was the rate of reconstructive failure, defined as complete autologous flap loss.

## Methods

### Study Design and Participants

This was an investigator-initiated, single-center, phase 2 clinical feasibility trial conducted at The University of Texas MD Anderson Cancer Center; patients were recruited between August 3, 2018, and August 2, 2022. The study was open to patients enrolled in a randomized clinical trial of HF vs CF RNI for invasive breast cancer (the Shortening Adjuvant Photon Irradiation [SAPHIRE] trial; ClinicalTrials.gov identifier: NCT02912312). The PreMRT group was a separate arm within the larger trial, and patients with a diagnosis of T0-T3, N0-N3b, M0 breast cancer requiring both mastectomy and RT and desiring IMBR were eligible. Before enrollment, patients were evaluated by a multidisciplinary team including a breast surgical oncologist, radiation oncologist, plastic surgeon, and a medical oncologist, to determine their suitability for the PreMRT treatment algorithm. Written informed consent was obtained from each participant; the study was approved by the University of Texas MD Anderson Cancer Center institutional review board, and analysis of the PreMRT cohort was approved by the data monitoring and safety board. Race and ethnicity were obtained by patient self-report and classified using the Office of Management and Budget minimum categories. Race and ethnicity were assessed to acknowledge potential disparities between the groups. The study followed the Consolidated Standards of Reporting Trials (CONSORT) reporting guideline and was conducted in accordance with the Declaration of Helsinki. Full details are provided in the trial protocol (Supplement 1).

### Procedures

PreMRT was planned to commence approximately 3 to 4 weeks after completion of neoadjuvant systemic therapy. Mastectomy and IMBR were performed at 2 to 6 weeks after completion of PreMRT, aiming for 3 (±1) weeks. The planned axillary surgery and need for axillary RT were determined preoperatively by the multidisciplinary team. Patients with cN0 disease underwent sentinel lymph node biopsy, and those with cN1 disease were considered for targeted axillary lymph node dissection. The RT treatment target was the breast and undissected lymphatics; the level I to II axilla, level III axilla, supraclavicular, and internal mammary lymph nodes (first 3 interspaces) were included as clinically indicated. The prescription dose for patients assigned to the HF-RT arm was 40.05 Gy in 15 fractions to the breast and 37.5 Gy in 15 fractions to the undissected lymphatics. For patients assigned to the CF-RT arm, the prescription dose was 50 Gy in 25 fractions to the breast and 45 Gy in 25 fractions to the undissected lymphatics. No breast boost was delivered.

At the time of mastectomy, patients underwent IMBR with microvascular transfer of an autologous tissue flap—deep inferior epigastric artery perforator (DIEP) or profunda artery perforator flap—or use of a pedicled latissimus dorsi flap (with or without an adjunctive prosthesis) or placement of a TE alone.

### Outcomes

The primary outcome was the rate of reconstructive failure, defined as complete autologous flap loss. Secondary outcomes included (1) number of patients who developed lymphedema within 24 months of RNI, (2) reconstructive complications, and (3) patient-reported quality of life. Mastectomy skin flap necrosis (MSFN) was defined as nonviable breast skin and graded using the validated SKIN (Skin Ischemia Necrosis) score.^28^ Any intraoperative surgical technical issue noted was included in the operative report. Major complications were defined as those that required hospital readmission, unplanned reoperation, or treatment with intravenous antibiotics or resulted in a delay of adjuvant therapy (>8 weeks).^29,30^ Surgical complications were categorized according to the Clavien-Dindo classification.

Time to locoregional recurrence and distant metastasis, disease-free survival, and overall survival were recorded. Locoregional recurrence was defined as disease recurrence in the chest wall and/or regional lymph nodes. Disease-free and overall survival were defined from the date of diagnosis. The residual cancer burden index was calculated using an online calculator.^31,32^

Patients underwent standardized evaluations before surgery and then at 3, 6, 12, 18, and 24 months after RT. These evaluations included standardized differences in volumes between the affected and unaffected arms using a perometer, with clinical lymphedema defined as a relative percentage difference of 10% or more reported on at least 1 occasion. Arm function and shoulder function were evaluated using the Disabilities of Arm, Shoulder and Hand questionnaire (QuickDASH-9)^33^ and the questions on the Functional Assessment of Cancer Therapy–Breast +4 (FACT-B+4) arm symptom subscale.^34^ A Satisfaction with Cosmetic Outcome Questionnaire was also completed.^35^ Radiotherapy-related skin and soft tissue toxic effects were evaluated using the Common Terminology Criteria for Adverse Events (CTCAE; version 4.0)^36^ scale during the final week of RT; at 6, 18, and 30 months after RT; and then every 12 months.

### Statistical Analysis

Analysis was performed on an intent-to-treat basis. Patients were randomized 1:1 to either CF or HF RNI before surgery using permuted block randomization with a block size of 4. As this was a feasibility study, the initial sample size was 30 patients, with interim analysis after 15 cases; subsequent cohort expansion to 50 patients was planned once the treatment sequence was demonstrated to be safe and feasible based on the predefined outcome parameter of reconstructive surgical procedures that experienced complete flap failure (<30%) and was approved by the institutional review board. Owing to a high reconstructive failure rate among patients who underwent TE reconstruction (50%), the protocol was amended to exclude these patients in the cohort expansion, effective August 19, 2021.

Patient characteristics were summarized using descriptive statistics. Distributions of continuous variables were summarized in terms of median (IQR) or mean (SD) values. Distributions of categorical variables were summarized in terms of frequency and percentage. Continuous variables were compared between groups by use of the Wilcoxon rank sum test, and associations between categorical variables were assessed using Fisher exact test. Variables with *P* < .05 under univariate analysis were considered in multivariable logistic regression analysis. All statistical evaluations were 2-sided, with *P* < .05 considered statistically significant; 95% CIs were computed using the exact binomial calculation. Statistical analysis was performed with SAS software, version 9.4 (SAS Institute Inc).

## Results

### Participants and Presurgical Treatment

Fifty women enrolled, of whom 48 underwent IMBR. One patient chose to leave the trial after randomization and is not included in the analysis, and 1 patient opted for delayed reconstruction after receiving PreMRT (Figure). One patient was randomized to receive CF-RT but received HF-RT owing to COVID-19–related service disruption.

**Figure.**
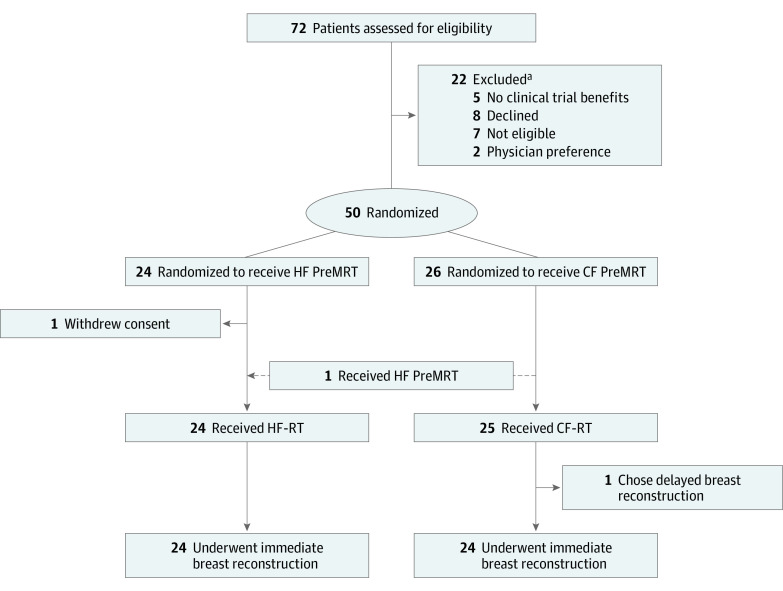
CONSORT Diagram for the Phase 2 Trial of Premastectomy Radiotherapy (PreMRT) CF indicates conventionally fractionated; HF, hypofractionated; and RT, radiotherapy. ^a^Screening data are unavailable for the time period of the study when the first 20 patients were enrolled.

Demographic and baseline clinical characteristics are summarized in Table 1. The median age was 48 years (range, 31-72 years), and the mean (SD) body mass index (BMI; calculated as weight in kilograms divided by height in meters squared) was 30 (7). All patients had unilateral breast cancer; most tumors were invasive ductal carcinoma (39 [80%]), and most were hormone receptor positive and ERBB2 negative (38 [78%]).

**Table 1.** Demographic and Baseline Clinical Characteristics of Patients Receiving Premastectomy RT

Characteristic	Patients, No. (%)		
	HF-RT group (n = 24)	CF-RT group (n = 25)	Total (N = 49)
Age at diagnosis, median (range), y	47 (31-63)	50 (33-72)	48 (31-72)
BMI, mean (SD)	30 (7)	30 (6)	30 (7)
Race and ethnicity			
Asian	0	2 (8)	2 (4)
Black or African American	3 (13)	0	3 (6)
White	19 (79)	19 (76)	38 (78)
Other^a^	0	1 (4)	1 (2)
Not reported	2 (8)	4 (16)	6 (12)
Ethnicity			
Hispanic or Latino	3 (13)	5 (20)	8 (16)
Not Hispanic or Latino	19 (79)	16 (64)	35 (71)
Not reported	2 (8)	4 (16)	6 (12)
Comorbidities			
Diabetes	1 (4)	1 (4)	2 (4)
Hypertension	5 (21)	6 (24)	11 (22)
Hyperlipidemia	2 (8)	10 (40)	12 (24)
Hypothyroidism	2 (8)	3 (12)	5 (10)
Cigarette smoking			
Current smoker	0	0	0
Previous smoker	7 (29)	3 (12)	10 (20)
Histologic subtype			
Invasive ductal	18 (75)	21 (84)	39 (80)
Invasive lobular	3 (13)	2 (8)	4 (8)
Mixed invasive ductal or lobular	2 (8)	2 (8)	5 (10)
Other	1 (4)	0	1 (2)
Clinical T category			
T1	2 (8)	2 (8)	4 (8)
T2	7 (29)	12 (48)	19 (39)
T3	15 (63)	11 (44)	26 (53)
Clinical N category			
N0	0	5 (20)	5 (10)
N1	23 (96)	17 (68)	40 (82)
N2	1 (4)	1 (4)	2 (4)
N3	0	2 (8)	2 (4)
Multifocal and/or multicentric disease	20 (83)	20 (80)	40 (82)
Receptor status			
ER and/or PR positive and ERBB2 negative	22 (92)	16 (64)	38 (78)
ERBB2 positive	2 (8)	7 (28)	9 (18)
Triple-negative breast cancer	0	2 (8)	2 (4)
Residual cancer burden index class^b^			
0/pCR	3 (13)	6 (24)	9 (21)
I	1 (4)	4 (16)	5 (11)
II	11 (46)	9 (36)	20 (46)
III	7 (29)	3 (12)	10 (23)

Abbreviations: BMI, body mass index (calculated as weight in kilograms divided by height in meters squared); CF, conventionally fractionated; ER, estrogen receptor; HF, hypofractionated; pCR, pathologic complete response; PR, progesterone receptor; RT, radiotherapy.

^a^ American Indian or Alaska Native.

^b^ Where able to be calculated (n = 44): 0, pathologic complete response (ypT0/isN0); I, minimal residual disease; II, moderate residual disease; and III, extensive residual disease.

Treatment is summarized in Table 2. Forty-six patients (94%) received neoadjuvant systemic therapy before PreMRT. Twenty-five patients received CF-RT, and 24 patients received HF-RT. The RT technique was volumetric modulated arc therapy for 18 patients (37%), a matched photon-electron technique for 23 (47%), and a partially wide tangent for 8 (16%). All patients received RNI that included the internal mammary nodes. Two patients (4%) received an internal mammary node boost, and 3 (6%) received an infraclavicular lymph node boost (10-16 Gy). No patient received a boost to the primary breast tumor.

**Table 2.** Treatment Details

Treatment	No. (%) (N = 49)
Neoadjuvant systemic therapy	46 (94)
Premastectomy radiotherapy to the breast	
50 Gy in 25 fractions	25 (51)
40.05 Gy in 15 fractions	24 (49)
Radiotherapy technique	
Matched photon-electron	23 (47)
Volumetric modulated arc therapy	18 (37)
Partially wide tangent	8 (16)
Regional nodal irradiation	
Internal mammary nodes	49 (100)
Supraclavicular fossa or axillary apex	49 (100)
Level I and II axilla	45 (92)
Regional nodal boost	
Infraclavicular fossa	3 (6)
Internal mammary nodes	2 (4)
None	46 (94)
Mastectomy type	
Skin sparing	44 (90)
Nipple sparing	4 (8)
Total^a^	1 (2)
Unilateral	32 (65)
Bilateral	17 (35)
Axillary lymph node surgery	
Axillary lymph node dissection	40 (82)
After upfront SLNB or TAD^b^	3 (6)
SLNB or TAD	9 (18)
Upfront^b^	4 (8)
Reconstruction type (n = 48)	
Microvascular autologous flap reconstruction	41 (85)
DIEP or MS-TRAM flap	37 (77)
PAP flap	4 (8)
Bipedicled DIEP or stacked DIEP or PAP flap	8 (17)
Latissimus dorsi flap	5 (10)
With adjunctive implant or tissue expander	4 (8)
Tissue expander (subpectoral)	2 (4)

Abbreviations: DIEP, deep inferior epigastric artery perforator; MS-TRAM, muscle-sparing transverse rectus abdominis myocutaneous; PAP, profunda artery perforator; SLNB, sentinel lymph node biopsy; TAD, targeted axillary lymph node dissection.

^a^ This patient did not undergo immediate breast reconstruction.

^b^ Upfront SLNB or TAD was done prior to initiation of premastectomy radiotherapy; if residual disease was present, a completion axillary lymph node dissection was performed at the time of mastectomy.

### Safety of the PreMRT Treatment Sequence

Mastectomy with IMBR was completed for 48 patients, at a median of 23 days (IQR, 20-28.5 days) after completion of RT. Thirty-seven of 48 patients (77%) underwent surgery within a mean (SD) of 3 (1) weeks after RT completion, and 47 underwent surgery within 6 weeks after RT completion. One patient experienced prolonged RT-related toxic effects (grade 2) that resulted in surgical delay and later received a diagnosis of pyoderma gangrenosum. Twenty-nine patients had CTCAE grade 1 dermatitis, and 10 patients had grade 2 dermatitis at completion of RT (grade 2: 1 of 24 [4%] in the HF-RT group vs 9 of 25 [36%] in the CF-RT group; *P* = .02). No patient had grade 3 or 4 RT-related toxic effects or had to discontinue RT.

Of the 48 patients who underwent IMBR, 44 (92%) had skin-sparing mastectomy, and 4 (8%) had nipple-sparing mastectomy (Table 2). Forty-one patients (85%) had microvascular autologous flap reconstruction, 5 patients (10%) had reconstruction with a pedicled latissimus dorsi flap, and 2 patients (4%) underwent TE placement. Two intraoperative events (5%) occurred during the 41 microvascular reconstructions that were successfully revised, 1 of which required conversion to thoracodorsal recipient vessels.

Regarding the primary end point, there were no complete autologous flap losses (Table 3). Overall, 10 of 48 patients (21%) had major surgical postoperative complications, 8 (17%) of whom had Clavien-Dindo classification grade 3b complications, including 2 partial flap losses, and 2 (4%) of whom experienced a delay in initiation of planned adjuvant therapy due to postoperative complications. The median interval between completion of neoadjuvant systemic therapy and surgery, including surgery for postoperative complications, was 11.7 weeks (IQR, 10.8-14 weeks). Both patients with TEs had infective complications (grade 3b), 1 of whom required explantation (50%). Mastectomy skin flap necrosis occurred in 8 of 48 patients (17%; 1 grade 3b). In 2 of the 17 bilateral cases, the MSFN occurred on the treated side, and in 1 of the 16 bilateral cases, the necrosis was bilateral.

**Table 3.** Recipient Site Complications and Revision Surgery Among Patients Who Underwent Premastectomy Radiotherapy and Immediate Breast Reconstruction

Complication or surgery	Cases, Total No. (%) (N = 48)
Complication	
Mastectomy skin flap necrosis	8 (17)
Delayed wound healing	1 (2)
Complete flap failure	0
Partial flap failure	2 (4)
Explantation of tissue expander	1 (2)
Flap fat necrosis	2 (4)
Hematoma	0
Seroma	3 (6)
Surgical site infection or cellulitis^a^	5 (10)
Additional surgical procedure for complication, No.	
1	7 (15)
≥2	1 (2)
Elective revision surgical procedure in breast area, No.	
1	28 (58)
≥2	8 (17)

^a^ Requiring oral or intravenous antimicrobial therapy.

On post hoc analysis, the postoperative complication rate was higher when surgery was performed more than 30 days after RT completion; however, this difference was not statistically significant (56% [5 of 9] vs 27% [10 of 37]; *P* = .13). Six of 48 patients (13%) received a diagnosis of clinical lymphedema, at a mean (SD) of 8.3 (3.2) months after RT completion.

### Variables Associated With Complications

On univariate analysis, older age and higher BMI were associated with complications (eg, complications requiring reoperation) among 46 patients who underwent autologous tissue IMBR (Table 4). There were no active cigarette smokers, and previous cigarette smoking was not associated with complications.

**Table 4.** Analysis of Variables Associated With Recipient Site Complications Among Patients Who Underwent Premastectomy Radiotherapy and Immediate Autologous Breast Reconstruction (N = 46)

Univariate analysis			Multivariate analysis	
Variable	OR (95% CI)	*P* value	OR (95% CI)	*P* value
**Any postoperative complication**				
Older age, y	1.09 (1.01-1.17)	.02	1.09 (1.01-1.18)	.04
Higher BMI	1.19 (1.05-1.36)	.009	1.21 (1.05-1.39)	.01
**Major postoperative complication**				
Older age, y	1.15 (1.03-1.28)	.07	1.23 (1.04-1.45)	.02
Higher BMI	1.17 (1.01-1.36)	.02	1.32 (1.03-1.70)	.03
**Complication requiring surgical procedure**				
Older age, y	1.16 (1.03-1.30)	.01	1.26 (1.03-1.55)	.03
Higher BMI	1.17 (1.00-1.37)	.03	1.37 (1.01-1.85)	.04

Abbreviations: BMI, body mass index; OR, odds ratio.

On multivariate analysis, older age (odds ratio [OR], 1.09; 95% CI, 1.01-1.18; *P* = .04) and higher BMI (OR, 1.21; 95% CI, 1.05-1.39; *P* = .01) were associated with any complications. Older age (OR, 1.26; 95% CI, 1.03-1.55; *P* = .03) and higher BMI (OR, 1.37; 95% CI, 1.01-1.85; *P* = .04) were also associated with complications requiring reoperation (Table 4).

### Recurrences, Survival, and Pathologic Response

There were no local, regional, or distant recurrences, and no patient had died at a median follow-up of 29.7 months (range, 10.1-65.2 months to last follow-up). Seven of 49 patients had a breast and axillary pathologic complete response (pCR) (ypT0N0), and 2 patients had only residual ductal carcinoma in situ (ypTisN0). The rate of pCR was higher in triple-negative and ERBB2-positive cancers than in luminal-type estrogen receptor–positive, ERBB2-negative cancers (45% [5 of 11] vs 5% [2 of 38]; *P* < .001).

### Patient-Reported Outcomes

At 6 and 12 months after RT completion, the HF-RT and CF-RT groups had similar mean (SD) scores on the QuickDASH-9 (14.7 [14.1] vs 20.1 [10.3]; *P* = .21; overall mean [SD] score, 17.4 [12.5]), FACT-B+4 arm symptom subscale (42.9 [12.5] vs 46.3 [9.2]; *P* = .38; overall mean [SD] score, 44.6 [10.9]), and Satisfaction with Cosmetic Outcome Questionnaire (68.3 [16.7] vs 61.3 [14.6]; *P* = .19; overall mean [SD] score, 64.8 [15.9]). At 18 and 24 months after RT completion, the HF-RT and CF-RT groups had similar mean (SD) scores on the QuickDASH-9 (11.5 [15.0] vs 22.5 [13.5]; *P* = .07; overall mean [SD] score, 16.3 [15.1]) and FACT-B+4 (41.0 [11.4] vs 46.1 [12.6]; *P* = .31; overall mean [SD] score, 43.2 [12.0]); however scores from the Satisfaction with Cosmetic Outcome Questionnaire were significantly better in the HF-RT group compared with the CF-RT group (72.4 [20.3] vs 56.4 [11.2]; *P* = .02; overall mean [SD] score, 65.3 [18.5]). The questionnaire completion rate was 77% (37 of 48) for the 6- and 12-month time points and 64% (25 of 39) for the 18- and 24-month time points.

### PMRT Cohort

During the same study period, 290 patients enrolled in the PMRT cohort of the SAPHIRE trial and underwent mastectomy: total mastectomy for 147 patients (51%), skin-sparing mastectomy for 123 patients (42%), and nipple-sparing mastectomy for 20 patients (7%). Of these, 142 patients (49%) underwent immediate TE placement, and 1 patient underwent immediate implant reconstruction; no patient underwent immediate autologous tissue flap reconstruction.

Regarding final outcomes, overall, 137 patients (47%) had not achieved breast reconstruction: 108 patients (37%) did not undergo any form of breast reconstruction, 9 patients (3%) had TEs in place but had not yet undergone definitive reconstruction, and 20 patients (7%) had explantation of a TE or implant and did not undergo subsequent reconstruction.

Definitive breast reconstruction was performed at a median of 12.2 months (IQR, 9.7-16.3 months) after mastectomy. Microvascular autologous tissue reconstruction was performed for 107 patients (37%) (including 1 stacked reconstruction), 14 patients (5%) received pedicled latissimus dorsi flaps (13 with an adjunctive implant), and 31 patients (11%) underwent final implant reconstruction.

Clavien-Dindo grade 3b complications occurred among 42 patients (15%), similar to the rate of 17% in the PreMRT cohort (*P* = .74). There were no complete flap failures, and 28 of 142 patients (20%) had explantation of TE for complications.

## Discussion

In this phase 2 randomized clinical trial of a new PreMRT sequence approach to RT delivery in breast cancer to facilitate IMBR, to our knowledge, the first in the US and the largest study of its kind, we found that microvascular autologous tissue reconstruction could be performed safely with good cosmetic outcomes while avoiding the risk of adjuvant RT treatment delays. All patients who underwent PreMRT received RNI that included the internal mammary lymph nodes, with 25 of 49 patients receiving CF-RT, which is the standard of care in the US for breast RT for patients with node-positive disease or large tumors.^37^ The internal mammary recipient vessels were used successfully in 98% (40 of 41) of the microvascular breast reconstructions with no complete flap losses. This finding contrasts with a recent prospective nonrandomized feasibility study from the Primary Radiotherapy And DIEP Flap (PRADA) trial group in the UK, where all patients received HF-RT and only 36% of patients received internal mammary nodal irradiation.^25^ The present study therefore establishes the safety of PreMRT in the setting of RNI that includes the internal mammary lymph nodes.

PreMRT enables patients to undergo definitive IMBR with its many inherent advantages while avoiding the negative late effects of RT on definitive breast reconstruction,^1,2,7,38,39^ even with HF-RT regimens,^40,41,42^ as well as avoiding the need for TE placement and the consequent risk of explantation (which occurred in 19% of patients in the PMRT cohort).^43,44,45^ Direct-to-implant reconstruction, although associated with acceptable outcomes in this setting, was only performed for 1 patient in the PMRT group.^43,46^ PreMRT also has the potential to increase the number of patients who undergo breast reconstruction; of the patients who received conventional PMRT, approximately 45% did not undergo definitive breast reconstruction, and among those who did, the median delay from mastectomy to reconstruction was approximately 1 year. However, all patients in the PreMRT arm desired, and were appropriate candidates for, breast reconstruction.

To our knowledge, few studies of PreMRT followed by breast reconstruction have been conducted, and most used whole-breast CF-RT with a dose of 50 Gy and pedicled flap or implant reconstruction.^18,20,21,22,41,47,48,49,50^ The PRADA trial, in which 33 patients underwent preoperative HF-RT (either 40 Gy in 15 fractions or 42.72 Gy in 16 fractions) followed by skin-sparing mastectomy and microvascular DIEP flap IMBR, reported no complete flap losses or serious treatment-related adverse events, with complication rates comparable to previous studies of PMRT.^25^ There were no locoregional recurrences, 4 patients (12%) developed distant metastatic disease, and 2 (6%) died from breast cancer at a median follow-up of 23.6 months. In the present study, there were no locoregional recurrences, no cases of distant metastasis, and no deaths from breast cancer during a median follow-up of 29.7 months (IQR, 25.5-40.1 months).^19,25,51^

The rate of MSFN in the present study (17%) is in keeping with rates in previous studies of PreMRT^20,21,22,49,50^ and comparable to rates reported with mastectomy and IMBR followed by standard PMRT.^20,50,51,52,53^ Most cases of MSFN in our study (7 of 8) were managed conservatively because there was no concern about delay of adjuvant RT. The incidence of major surgical postoperative complications (21%) also compares favorably with other studies—the prospective multicenter Mastectomy Reconstruction Outcomes Consortium reported reoperative complications in 29.2% of patients and reconstructive failure in 1.3% of patients who received DIEP flaps.^54^ The complication rates in the present study were similar between patients who received HF-RT and patients who receuved CF-RT. However, there was a higher incidence of CTCAE grade 2 dermatitis, and patient-reported satisfaction with cosmetic outcome at 18 and 24 months was lower, among the CF-RT group.

### Limitations

This study has some limitations. The main limitation is that the PreMRT randomized phase 2 component of this trial was underpowered to reliably compare different RT fractionation schedules or evaluate oncologic outcomes, and the RT delivery techniques were not standardized; the response rate for the patient-reported outcomes may also result in study bias. This was a feasibility trial, however, and the outcomes aided in the development of a recently launched subsequent larger clinical trial in which 126 patients are randomized to receive HF-PreMRT or CF-PreMRT followed by mastectomy and autologous tissue IMBR (Trial of Preoperative Radiation [TOPAz]; ClinicalTrials.gov identifier: NCT05774678, activated April 5, 2023). A concern regarding PreMRT is the potential to result in a local pCR among patients who would otherwise not have attained pCR from neoadjuvant systemic therapy alone.^18^ This scenario could lead to some patients not receiving evidence-based adjuvant therapies reserved for patients with less than pCR after neoadjuvant systemic therapy. To address this concern, in the present randomized clinical trial (NCT05774678), patients received a further tumor core biopsy after neoadjuvant systemic therapy and before PreMRT.

## Conclusions

In this randomized clinical trial study of PreMRT for breast cancer with RNI including the internal mammary nodes, the first such trial in the US to our knowledge, we have demonstrated that this treatment sequence is feasible and safe in terms of complications and locoregional control. This innovative therapeutic sequence, which is now undergoing further investigation, allows patients to receive the advantages of IMBR, including shortening the overall time for the breast reconstructive process, thus potentially increasing the number of patients who undergo postmastectomy breast reconstruction.

## Notes

Supplement 1.
Trial Protocol

Supplement 2.
Data Sharing Statement
