# Supplementary material for: Premastectomy Radiotherapy and Immediate Breast Reconstruction: A Randomized Clinical Trial
Source: JAMA Netw Open. 2024 Apr 5;7(4):e245217. doi: 10.1001/jamanetworkopen.2024.5217 (PMC10998161; doi:10.1001/jamanetworkopen.2024.5217)
Supplement: Supplement 1. — Trial Protocol [file jamanetwopen-e245217-s001.pdf]

Shortening Adjuvant PHoton IRradiation (SAPHIRe): A Randomized Trial of Hypofractionated Versus Conventionally Fractionated Regional Nodal Irradiation for Invasive Breast Cancer

Principal Investigator

Karen Hoffman, MD, MHSc, MPH

The University of Texas MD Anderson Cancer Center

Department of Radiation Oncology

1515 Holcombe Blvd, Unit 1202

Houston, Texas 77081

713-563-2339

Khoffman1@mdanderson.org

Schema

<u>Lymphedema cohort</u> (MD Anderson Cancer Center and Houston- Area Locations will enroll patients into this cohort and will follow the Schedule of Events listed in section 8.4.8. Cancer Network Sites and Orlando Health are not participating in this cohort.)

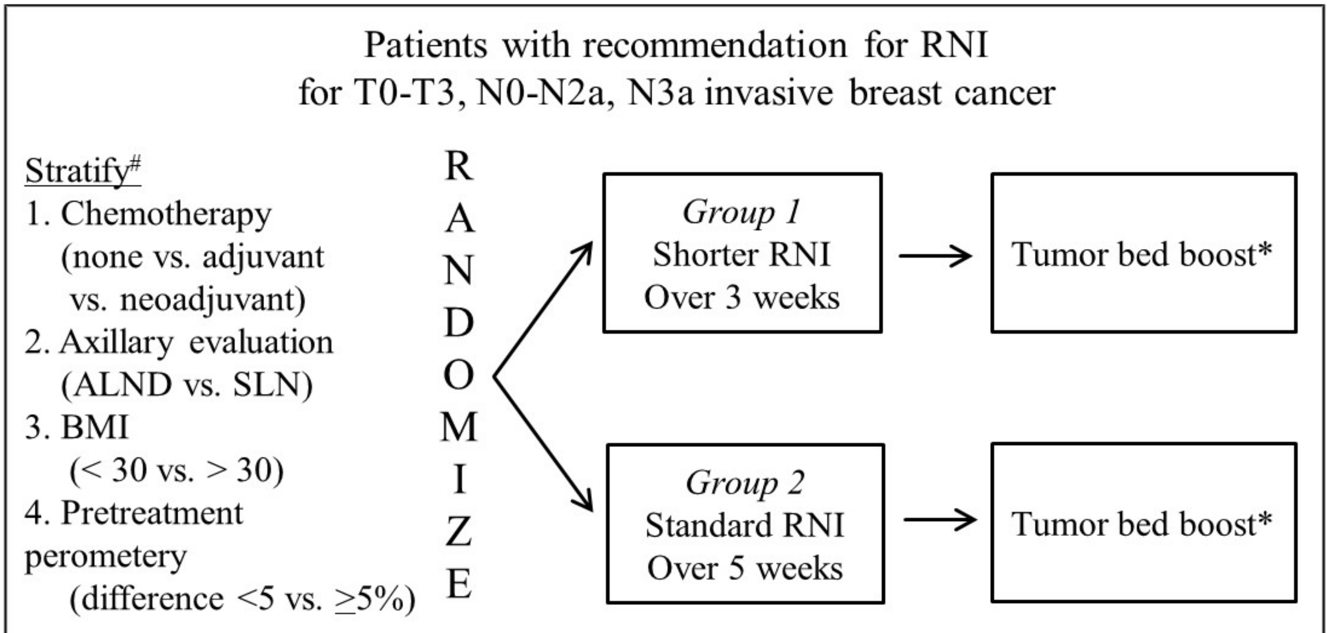

Abbreviations: RNI=Regional nodal irradiation, delivered as a component of post mastectomy radiation therapy for patients who had mastectomy or delivered with whole breast radiation for patients who had breast conserving surgery. ALND=Axillary lymph node dissection. SLN=sentinel lymph node evaluation. BMI=Body mass index.
#Patients in the initial trial cohort to evaluate arm lymphedema are also stratified by difference in arm volume prior to RNI (<5 vs. >5%).
*Chest wall boost for patients who had mastectomy; resection cavity boost for patients who had breast conserving surgery.

Locoregional control cohort (MD Anderson Cancer Center, Houston Area Locations, participating MD Anderson Network Sites, and Orlando Health will enroll patients in this cohort. MD Anderson Cancer Center and Houston Area Locations will follow the Schedule of Events listed in section 8.4.8. Only participating MD Anderson Network Sites and Orlando Health will follow the Schedule of Events listed in section 8.4.9.)

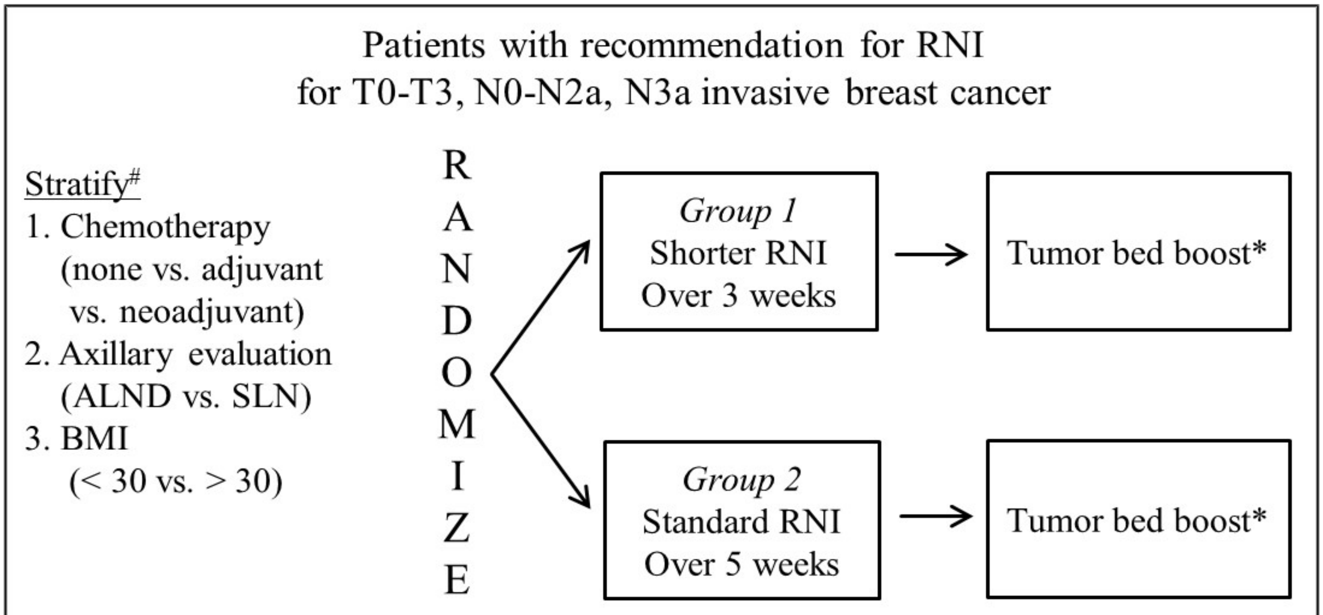

Abbreviations: RNI=Regional nodal irradiation, delivered as a component of post mastectomy radiation therapy for patients who had mastectomy or delivered with whole breast radiation for patients who had breast conserving surgery. ALND=Axillary lymph node dissection. SLN=sentinel lymph node evaluation. BMI=Body mass index.
[#]Patients in the initial trial cohort to evaluate arm lymphedema are also stratified by difference in arm volume prior to RNI (<5 vs. >5%).
*Chest wall boost for patients who had mastectomy; resection cavity boost for patients who had breast conserving surgery.

1 BACKGROUND

1.1 Frequency and morbidity of lymphedema

Regional nodal irradiation (RNI) is a standard component of postmastectomy radiation therapy (PMRT) and is delivered to many women who receive whole breast radiation for Stage II or III breast cancer. Although RNI improves breast cancer control and survival, it also increases the risk of developing lymphedema after breast cancer treatment (Erickson 2001; Shah 2012; Warren 2014). It is estimated that up to 60% of women treated with mastectomy, axillary lymph node dissection, and PMRT develop lymphedema (Shah 2012). In a previous clinical trial we found 30% of women treated with mastectomy, axillary lymph node dissection, and PMRT at MD Anderson developed lymphedema (Woodward 2008). We believe the frequency of lymphedema at our institution is higher than some series due to higher prevalence of lymphedema risk factors, including a 50% prevalence of obesity in our radiated patients (Shaitelman 2015) and more extensive axillary surgery performed by our surgeons. Most lymphedema is apparent within two years of surgical treatment (Wetzig 2014).

Arm swelling from lymphedema can be extremely debilitating, causing pain, limiting arm movement, limiting work, and limiting what clothes a woman can wear (Erickson 2001; McWayne 2010). Not surprisingly, breast cancer patients who develop lymphedema experience more psychological distress, report poorer quality of life, and are more likely to be disabled than those who do not develop lymphedema (Erickson 2001; McWayne 2010). Given the morbidity of lymphedema, it is important to develop interventions that decrease the risk of lymphedema after breast cancer treatment without sacrificing local-regional control or survival.

1.2 Toxicity benefits of shorter-course radiation

Randomized trials with ten years of follow up demonstrate shorter-course radiation to the intact breast provides equivalent cancer control while reducing radiation side effects, compared to longer-course radiation (Haviland 2013; Whelan 2010). In the START B trial, patients who received 40 Gy in 15 fractions over three weeks were less likely to develop breast edema (HR 0.55 [95% CI 0.39-0.79]; p=0.001), telangiectasia (HR 0.62 [95% CI 0.40-0.96]; p=0.001), and breast shrinkage (HR 0.80 [95% CI 0.67-0.96]; p=0.015), than patients who received 50 Gy in 25 fractions over five weeks (Haviland 2013). A trial recently completed by our breast radiation oncology group that compared shorter versus longer radiation to the intact breast (MD Anderson

2010-0559), similarly demonstrated shorter radiation decreased acute dermatitis and breast pain during radiation and decreased both physician- and patient-reported fatigue at six months after completion of radiation (Shaitelman 2015). Shorter-course radiation is now standard treatment for women receiving radiation therapy to the intact breast only (Smith B 2011), but this approach is not used for delivery of RNI with breast radiation or for delivery of RNI as a part of PMRT in the United States (US).

Shorter-course radiation has not been well studied in the setting of RNI. The randomized trials evaluating shorter-course radiation to the intact breast did enroll some patients who received PMRT or radiation therapy to the regional-nodal basins in addition to the intact breast. The START A trial (comparing 50 Gy in 25 treatments versus 41.6 Gy in 13 treatments versus 39 Gy in 13 treatments, all delivered over 5 weeks) enrolled 318 patients who received RNI. At a median follow up of 9.3 years there was no difference in shoulder stiffness and a nonsignificant decrease in arm edema in women who received 39 Gy (HR 0.50 [95% CI 0.20-1.30]; p=0.16) (Haviland 2013). The START B trial enrolled 161 patients who received RNI and compared 50 Gy in 25 treatments over five weeks to 40 Gy in 15 treatments over three weeks, the treatment regimens that will be used in our trial. At a median follow up of 9.9 years there was no difference in shoulder stiffness and a nonsignificant decrease in arm edema in women who received 40 Gy (HR 0.42 [95% CI 0.11-1.63]; p=0.21) (Haviland 2013). It is possible there was underascertainment of upper extremity lymphedema in the START trials because there were no volumetric measurements of upper extremity size, and upper extremity lymphedema was not a primary endpoint. It is notable that the proportional reduction in arm edema (HR 0.42) in the subgroup of patients who received RNI is similar to the statistically significant proportional reduction in breast edema in the larger study cohort (HR 0.55). A small randomized trial from Brussels (50 Gy in 25 fractions versus 42 Gy in 15 fractions) included 24 patients who received RNI and found that the shorter regimen reduced skin changes and had less impact on lung function (Van Parijs 2012). In total, the data from the randomized trials of short-course radiation to the intact breast support the hypothesis that short-course RNI may reduce lymphedema and skin and tissue toxicity.

1.3 Rationale for reduced edema with shorter-course radiation

How larger daily doses of radiation coupled with a lower total dose may reduce the risk of lymphedema is not known. Some believe that the biologic equivalent dose delivered with regimens that deliver larger daily doses of radiation is simply slightly lower than the biologic equivalent dose delivered with standard doses and that this slightly lower biologic dose explains the reduction in side effects. Others believe that there is a not yet understood biologic basis for the reduction in side effects. We postulate the reduction in lymphedema risk after larger daily doses of radiation is the consequence of a shift in cytokine profiles. It is well recognized that immune surveillance reflects a balance between pro-inflammatory, anti-tumor roles and anti-inflammatory, pro-tumor roles. This balance has been characterized by the cytokines secreted by Th1 anti-tumor immune cells versus Th2 pro-tumor immune cells. Several studies demonstrate that very large daily doses of radiation stimulate Th1 production (Gough 2010; Lee, 2009; Reits 2006; Finkelstein 2011). The larger daily doses delivered with our shorter RNI regimen may similarly stimulate Th1 production. The balance of Th1 anti-tumor immune cells vs. Th2 pro-tumor immune cells has also been implicated in lymphedema development. The only published predictor of lymphedema identified six cytokines largely associated with a Th2 cytokine balance that were strongly upregulated in patients with lymphedema (Lin 2012; receiver operating characteristic curve of 87%). Therefore a shift in cytokine profiles towards more Th1 anti-tumor immune cells (vs. Th2 pro-tumor immune cells) may be the biologic mechanism behind reduced lymphedema after treatment regimens that deliver larger daily doses of radiation. It is important to evaluate if cytokine profiles predict lymphedema and to validate the only published predictor of lymphedema in a prospective breast cancer cohort.

1.4 Need to establish the cardiac safety of the shorter treatment regimen

Shorter-course radiation is now standard-of-care treatment for women receiving radiation therapy to the intact breast only (Smith B 2011). However, shorter-course intact breast radiation was not widely adopted until there was 10 year follow up data establishing the cardiac safety of the treatment regimen. Modeling studies demonstrate the shorter-course radiation regimen being evaluated in this study (40 Gy in 15 fractions) delivers lower biologically equivalent dose to

the heart than the standard regimen (50 Gy in 25 fractions). Therefore, the shorter regimen is expected to cause less cardiac harm (Appelt 2013). However, there is no prospective data evaluating the cardiac safety of shorter-course regional nodal radiation to confirm it does not cause cardiac damage.

Echocardiography with global longitudinal strain (GLS) measurements is an accepted tool for early detection of myocardial changes (Plana 2014). GLS reflects the deformation of the myocardium over the course of the cardiac cycle. Early changes in GLS, indicating subclinical cardiac injury after exposure to cardiotoxic agents, have been associated with subsequent, clinically relevant cardiac dysfunction (Mornos 2013, Negishi 2013, Sawaya 2012, Fallah-Rad 2011). A relative decline in GLS has been observed after RT in patients treated for left-sided breast cancers. This change was seen immediately after the completion of RT and persisted at 14 months. The mean left ventricular dose in these studies was 6.7 and 9 Gy. The decline in strain was limited to the anterior segments of the heart, which received the highest radiation dose (Erven 2011, Erven 2013). Serum biomarkers are a useful complement to echocardiographic studies. They have been used for early identification, assessment, and monitoring of cardiotoxicity from cancer therapy, including RT. Promising biomarkers include troponins, natriuretic peptides, and markers of inflammation and fibrosis, including myeloperoxidase (MPO), C-reactive protein (CRP), galectin-3 (Erven 2013, Nellessen 2010, Ky 2014, D'Errico 2012, Jingu 2007, Lipshultz 2012).

Therefore, echocardiography with GLS and cardiac biomarkers will be studied in a subset of enrolled patients at MD Anderson Cancer Center and Houston Area Locations to evaluate the cardiac safety of short-course regional nodal radiation. It is essential to establish the cardiac safety of shorter-course, hypofractionated radiation, to the regional nodal basins in this phase II study in order to enable subsequent development of this treatment strategy in a phase III trial.

1.5 Potential effects of homocysteine and cholesterol regulation

In a previous clinical trial at MD Anderson we found a correlation between the presence of elevated serum homocysteine levels and the incidence of lymphedema (Woodward 2008). Although elevated homocysteine levels have been implicated in a range of vascular diseases, it has not been linked to lymphedema incidence (McCully 2005). Hyperhomocysteinemia may

develop as a consequence of defects in the metabolizing genes, folate and vitamin B6/B12 deficiencies (Garcia-Tevijano 2001). It is unknown what interactions, if any, these factors have in the incidence of lymphedema. However, there is a need for further study of the possibility of a causal association (direct or indirect) between lymphedema and elevated serum homocysteine. If further investigation can confirm a tenable association between the two then it may be possible to lower the risk of lymphedema by modulating the serum homocysteine level with a preventative measure as simple as oral vitamin supplements.

Our collaborators recently found a provocative relationship between clinical outcome after breast cancer treatment (including local recurrence after local radiation therapy) and cholesterol regulation in women with inflammatory breast cancer that is replicated in preclinical studies (Lacerda 2014; Wolfe 2015). They found cholesterol manipulation impacts radiation resistance in cancer stem cell surrogate assays more significantly than virtually every other genetic approach explored. Most recently, they found specific cellular components of the microenvironment can promote radiation resistance and stemness, and that these effects can be blocked by manipulating cholesterol transport. Lipoproteins were also a significant prognostic factor in a large clinical dataset of triple negative breast cancer patients. Prospective collection of lipid panels and correlation of these lipid panels to clinical outcomes including local control will provide further insights.

1.6 Potential for DNA repair capacity to predict treatment toxicity.

Previously published studies in both breast and prostate cancer have demonstrated that the degree of chronic side effects from radiation therapy can be predicted by the accumulation and resolution of the γ-H2AX foci in cells exposed to ionizing radiation (vanOorschot 2014, Chua 2011). In prostate cancer patients, those who had persistent bladder and/or rectal toxicity after radiation therapy showed a higher level of detected residual γ-H2AX foci in peripheral lymphocytes compared to patients without chronic toxicity.(Chua 2011). We will evaluate our hypothesis that abnormal DNA repair capacity contributes to the presence of acute radiation therapy related toxicity and patient-reported QOL decrements such as fatigue and arm and shoulder symptoms in patients receiving RNI for breast cancer. This is not applicable for the Cancer Network Site patients or Orlando Health.

1.7 Convenience and cost benefits of shorter-course radiation

Patients who receive standard RNI must undergo daily treatments, Monday through Friday, that last for five to six weeks. Out-of-pocket expenses can add up over the multiple weeks of daily radiation treatment, including copayments, travel to appointments, childcare and eldercare expenses, and lost wages. The time and financial burden of daily treatments can take its toll on patients and their families and can cause psychological and financial distress. Many patients spend more money than expected on cancer care, incur medical debt that lasts several years after cancer treatment, and report the financial cost of cancer treatment causes distress (Jagsi 2014; Markman 2010). Reducing the length of RNI from five to six weeks to three to four weeks, would reduce a patient's time away from personal, work, and family obligations and would reduce out-of-pocket expenses. Analysis of insurance claim reimbursement for PMRT performed by our group demonstrates shortening treatment duration from five to three weeks reduces reimbursement by 20%. More important from a patient perspective is the expected reduction in out-of-pocket expenses. Given the time and financial burden of standard RNI, it is important to develop a less burdensome and less costly RNI regimen.

1.8 Survival benefits of RNI

Administration of PMRT, which includes RNI, improves breast cancer survival for women with lymph-node positive breast cancer, with randomized trials demonstrating a 6% improvement in breast cancer survival 10 years after treatment and 8% improvement in breast cancer survival 20 years after treatment (EBCTCG 2014). Despite the life-saving benefits of PMRT, many women forgo PMRT (Shirvani 2011). A randomized trial demonstrates the addition of RNI to whole breast radiation improves disease free survival in women with node-positive breast cancer with a trend toward improved overall survival (Whelan 2011).

Fear of developing radiation-induced lymphedema and the inconvenience and cost of receiving five to six weeks of daily radiation treatment are two reasons women report they do not receive radiation treatment (Jagsi 2009). Development of a shorter regimen that decreases the risk of developing lymphedema and decreases the burden and cost of treatment is expected to increase

the quality of life of breast cancer survivors and increase the use of RNI. The increased use of RNI will reduce breast cancer mortality.

1.9 Preoperative radiation therapy

The need for post-mastectomy radiotherapy (PMRT) has complicated the use of breast reconstruction. Traditionally, patients at MD Anderson needing post-mastectomy radiotherapy either do not have immediate breast reconstruction or undergo a temporary based tissue-expander procedure that has been associated with complete loss of the reconstruction in 32%, a 26% rate of wound healing (necrosis) issues, and the need for multiple delayed surgeries to complete the final reconstruction (Kronowitz 2011); A multidisciplinary protocol for planned skin-preserving delayed breast reconstruction for patients with locally advanced breast cancer requiring postmastectomy radiation therapy: 3-year follow-up. The relative timing of surgery and radiation therapy does not impact locoregional control (Riet 2017; Giacalone 2010; Ho 2012; Nestle-Kramling 2016; Otte 2016), or mastectomy skin flap- and autologous flap-related complications (Ho 2012; Paillocher 2016; Thiruchelvam 2017; Zinzindohoué 2016; Monrigal 2011). Preoperative radiation therapy presents an opportunity for patients to achieve immediate breast reconstruction at the time of mastectomy and this approach is gaining increased use based on contemporary reports of its safety (Riet 2017; Giacalone 2010; Ho 2012; Nestle-Kramling 2016; Otte 2016; Ho 2012; Paillocher 2016; Thiruchelvam 2017; Zinzindohoué 2016; Monrigal 2011). Patients would therefore only require one definitive oncological procedure and complete their reconstruction in one step with a single hospitalization and recovery period. The risk of infection and loss of reconstruction as well as potential of delay to RT, discomfort, and permanent chest wall deformation from using a tissue expander whilst awaiting definitive reconstruction are avoided. The psychosocial morbidity of living with an unreconstructed breast until breast reconstruction can be performed at one-year and longer following completion of PMRT is also abrogated for those that chose not to, or are not able to, have a tissue expander (Teo 2016; Baumann 2011). Most importantly, this strategy has the potential to increase the number of patients that complete breast reconstruction following mastectomy and safety data regarding this approach from several centers around the world demonstrate this to be a

potentially acceptable approach. Therefore, patients receiving preoperative radiotherapy will be eligible to participate in this trial as well and analyzed as a separate cohort.

1.10 The need for additional evidence to support the efficacy of hypofractionated RNI

Most ongoing randomized trials comparing shorter hypofractionated RNI to conventional RNI are designed to evaluate the toxicity and safety of hypofractionated RNI or the impact of hypofractionated RNI on complication and cosmetic outcomes in the context of breast reconstruction rather than oncologic outcomes (NCT02700386, NCT02384733, NCT02958774, NCT03127995, NCT03414970, and NCT03422003).

The one recently published trial showing similar cancer control after short-course RNI delivered as a component of postmastectomy radiation therapy has not changed practice patterns (Wang 2019). The trial has been critiqued for having a high rate of locoregional recurrence, recruiting from only one treatment center, using an unconventional hypofractionation schedule, using outdated two-dimensional radiotherapy techniques, not treating the internal mammary lymph nodes, and excluding patients who undergo breast reconstruction (Jagsi 2019, Marta 2019). Current National Comprehensive Cancer Network Guidelines recommend delivery of conventional, five to six weeks of radiation therapy, for patients receiving radiation therapy to the regional lymph nodes (45-50.4 Gy in 25-28 fractions to the regional nodes, NCCN Guidelines Version 4.2020) they do not list hypofractionation as a treatment option for regional nodal irradiation. Most patients receiving regional nodal irradiation for breast cancer in the United States and most of Europe receive conventional fractionation. Therefore, there is a critical need for high-level evidence supporting the efficacy of shorter hypofractionated RNI to change practice in many countries worldwide.

1.11 Clonal hematopoiesis and treatment toxicity

Clonal hematopoiesis (CH) is defined by somatic (i.e. acquired) mutations that result in the disproportionate expansion of mutated cell lineages in the hematopoietic system. Massively parallel DNA sequencing technologies have permitted identification of CH mutations in the blood of otherwise healthy individuals that represent the expansion of a single cell lineage, or clone.

This process has been identified as common in normal aging with the majority of older individuals harboring detectable CH mutations (Busque 1996; Jacobs 2012). Studies in patients without hematologic disease have found that CH mutations confer an increased risk of hematologic cancer as well as non-hematologic disease, including cardiometabolic and pulmonary disease (Buscarlet 2017; Jaiswal 2014). Existing studies suggest that CH may impact non-hematologic disease risk by augmenting inflammatory pathways (Fuster 2017), which may make individuals who carry these mutations more susceptible to radiation toxicity. More recently, CH mutations have been found to be common among individuals with solid malignancy and to be associated with worse overall survival (Coombs 2017). Retrospective studies suggest that CH may occur after/be associated with cancer therapies (e.g. radiation therapy, chemotherapy) and may interact with these therapies to result in adverse treatment side effects (Coombs 2017; Takahashi 2017). However, it is unknown whether individuals who carry CH mutations and are exposed to radiation therapy or chemotherapy, or who develop detectable CH mutations following therapy, are at an increased risk for adverse treatment effects (e.g. cardiac toxicity) and inferior oncologic outcomes.

2 STUDY HYPOTHESIS AND OBJECTIVES

2.1 INITIAL TRIAL COHORT TO EVALUATE ARM LYMPHEDEMA (Lymphedema Cohort)

2.1.1 Study Hypothesis

Our central hypothesis is that a shorter (3 week) RNI treatment regimen, compared to a standard (5 week) RNI treatment regimen, will reduce the risk of developing lymphedema after radiation treatment by 50%.

2.1.2 Primary Objective: To compare the risk of developing lymphedema, defined as a 10% difference in volume between the affected and unaffected arm, within 24 months of RNI between patients assigned to short versus standard RNI.

2.2 EXPANDED TRIAL COHORT TO EVALUATE LOCOREGIONAL CONTROL (Locoregional control cohort)

2.2.1 Study Hypothesis

Our central hypothesis is that a shorter (3 week) RNI treatment regimen, compared to a standard (5 week) RNI treatment regimen, will provide equivalent locoregional cancer control.

2.2.2 Primary Objective: To compare the risk of locoregional recurrence, defined as a cancer recurrence in the ipsilateral breast, chest wall, or regional lymph nodes (axillary levels I-III, supraclavicular, or internal mammary)

2.3 SECONDARY OBJECTIVES FOR BOTH COHORTS

1) To compare the maximum standardized difference in arm volume between the affected arm and the unaffected arm within 24 months after completion of RNI between the two treatment arms.
2) To compare maximal acute (within 6 weeks of treatment) and late (more than 6 weeks after treatment) skin and soft tissue toxicities using the NCI CTCAE v4.0 scale between patients assigned to short versus standard RNI.
3) To compare patient-reported arm and shoulder function for the two treatment arms using the QuickDASH-9.
4) To compare patient quality of life for the two treatment arms using EQ-5D-3L and PROMIS Fatigue SF 6a.
5) To compare the peripheral blood cytokine profile at the end of radiation between patients assigned to short versus standard RNI.
6) To evaluate the effect of the peripheral blood cytokine profile at the end of radiation on developing lymphedema. (optional blood draw for a subset of patients and not applicable for the participating Cancer Network Site patients, or Orlando Health)
7) To evaluate the effect of homocysteine levels prior to radiation on developing lymphedema. (optional blood draw for a subset of patients and not applicable for the participating Cancer Network Site patients, or Orlando Health)
8) To evaluate time to distant metastasis, disease-free survival and overall survival for patients assigned to short versus standard RNI.
9) To evaluate the effect of lipid profiles prior to radiation on acute toxicity, patient reported outcomes, and the time to locoregional recurrence. (optional blood draw for

a subset of patients and not applicable for the participating Cancer Network Site patients, or Orlando Health)

10) To evaluate if DNA repair capacity contributes to the presence of acute radiation related toxicity and patient-reported quality of life decrements including fatigue and arm and shoulder symptoms. (optional blood draw for a subset of patients and not applicable for the participating Cancer Network Site patients, or Orlando Health)
11) To evaluate changes in echocardiography global longitudinal strain measurements and cardiac serum biomarkers, based on measurements performed before and after RT, in patients assigned to short versus standard RNI. (optional procedure for a subset of patients and not applicable for the participating Cancer Network Site patients, or Orlando Health)
12) In a separate cohort of 50 patients who will receive RNI before surgery, to collect information on number of patients developing lymphedema within 24 months of RNI on reconstructive complications, and on patient reported quality of life, in those assigned to short versus standard RNI. Participating Cancer Network Sites, or Orlando Health, are not participating in this cohort.
13) To evaluate patient-reported financial burdens using the Economic Strain and Resilience in Cancer- Financial Well- Being instrument.
14) To determine whether radiation therapy induces de novo detectable clonal hematopoiesis mutations or alters the genetic landscape of existing clonal hematopoiesis mutations (optional blood draw for a subset of patients and not applicable for the participating Cancer Network Site patients, or Orlando Health).
15) To determine whether radiation therapy or chemotherapy among individuals with pre- and/or post-treatment clonal hematopoiesis mutations is associated with adverse treatment effects and worse oncologic outcomes (optional blood draw for a subset of patients and not applicable for the participating Cancer Network Site patients, or Orlando Health).

3 PATIENT SELECTION

3.1 Inclusion Criteria, if receiving postoperative radiation therapy

3.1.1 Radiation oncologist recommends radiation treatment to the supraclavicular and infraclavicular fossa (i.e. RNI).

3.1.2 Pathologically-confirmed invasive breast cancer. If patients undergo upfront surgery, the pathologic stage must be T0-T3, N0-N2a or N3a. If patients receive neoadjuvant chemotherapy prior to surgery, the clinical stage must be T0-T3, N0-N2a or N3a. T4b disease is permitted if the patient undergoes breast conserving surgery.

3.1.3 Treatment with mastectomy or segmental mastectomy and axillary evaluation (sentinel node evaluation, axillary sampling, or axillary lymph node dissection). If the patient has T0 disease, breast surgery is not required.

3.1.4 Age 18 years or older.

3.1.5 If enrolling on in the arm lymphedema assessment cohort, documentation of arm volume measurement by perometer prior to axillary surgery.

3.1.6 If the patient has a history of a prior non-breast cancer, all treatment for this cancer must have been completed prior to study registration, and the patient must have no evidence of disease for this prior non-breast cancer.

3.1.7 Patients must be enrolled on the trial within 24 weeks of the later of two dates: the final breast cancer surgical procedure or administration of the last cycle of cytotoxic chemotherapy. If after trial enrollment it is determined the patient requires additional cytotoxic chemotherapy or additional breast cancer surgery prior to radiation therapy the patient may stay on trial but the patient must start radiation therapy within 24 weeks of the final breast cancer surgical procedure or administration of the last cycle of cytotoxic chemotherapy.

3.2 Inclusion Criteria, if receiving preoperative radiation therapy

3.2.1 Surgeon and radiation oncologist recommend preoperative radiation therapy

3.2.2 Radiation oncologist recommends radiation treatment to the supraclavicular and infraclavicular fossa (i.e. RNI).

3.2.3 Pathologically-confirmed invasive breast cancer. The clinical stage must be T0-T3, N0-N3b

3.2.4 Planned treatment with mastectomy and axillary evaluation (sentinel node evaluation, axillary sampling, or axillary lymph node dissection).

3.2.5 Planned breast reconstruction with autologous reconstruction.

3.2.6 Age 18 years or older.

3.2.7 If the patient has a history of a prior non-breast cancer, all treatment for this cancer must have been completed prior to study registration, and the patient must have no evidence of disease for this prior non-breast cancer.

3.3 Exclusion Criteria, if receiving postoperative radiation therapy

3.3.1 Pathologic or clinical evidence for a stage T4 breast cancer. However, T4b disease is permitted if the patient undergoes breast conserving surgery.

3.3.2 Pathologic or clinical evidence for a stage N2b, N3b, or N3c breast cancer (supraclavicular, or internal mammary lymph node involvement).

3.3.3 Clinical or pathologic evidence for distant metastases.

3.3.4 Current diagnosis of invasive breast cancer in the contralateral breast (ductal carcinoma in situ is permitted).

3.3.5 Prior diagnosis of invasive breast cancer in the contralateral breast.

3.3.6 If enrolling on in the arm lymphedema assessment cohort, current diagnosis of bilateral breast cancer.

3.3.7 History of therapeutic irradiation to the breast, lower neck, mediastinum or other area in which there could potentially be overlap with the affected breast. Except those patients enrolled and treated on the PRECISE trial (MD Anderson 2017-0362).

3.3.8 Patient is pregnant.

3.3.9 Patients who are cognitively impaired. Subjects will undergo a brief physical exam including a brief exam to determine cognitive review.

3.4 Exclusion Criteria, if receiving preoperative radiation therapy

3.4.1 Pathologic or clinical evidence for a stage T4 breast cancer.

3.4.2 Pathologic or clinical evidence for a stage N3c breast cancer (supraclavicular lymph node involvement).

3.4.3 Clinical or pathologic evidence for distant metastases.

3.4.4 Current diagnosis of invasive breast cancer in the contralateral breast (ductal carcinoma in situ is permitted).

3.4.5 Prior diagnosis of invasive breast cancer in the contralateral breast.

3.4.6 History of therapeutic irradiation to the breast, lower neck, mediastinum or other area in which there could potentially be overlap with the affected breast. Except those patients enrolled and treated on the PRECISE trial (MD Anderson 2017-0362).

3.4.7 Patient is pregnant.

3.4.8 Patients who are cognitively impaired. Subjects will undergo a brief physical exam including a brief exam to determine cognitive review.

4 REGISTRATION PROCEDURE

4.1.1 Informed Consent

Informed consent must be obtained from each subject during the clinic visit or remotely (electronic informed consent) prior to enrolling the subject into the protocol. The method of obtaining and documenting the informed consent and the contents of the consent must comply with ICH-GCP and all applicable regulatory requirements. Informed consent for this study may only be obtained by the Principal Investigator or the assigned designee. This delegation will be included on a protocol delegation log that will be signed by the site's PI. All consented subjects must be registered in the clinical trials management system, OnCore. Informed Consent Forms for enrolled patients and for patients who are enrolled but not eligible to receive study treatment (screening failures) will be maintained at the study site. The investigator will maintain a screening log to record details of all patients screened and to confirm eligibility or record reasons for screening failure, as applicable.

The investigator is responsible for: ensuring that the patient understands the potential risks and benefits of participating in the study; ensuring that informed consent is given by each patient, this includes obtaining the appropriate signatures and dates on the ICF prior to the performance of any study procedures and prior to the administration of study treatment; answering any questions the patient may have throughout the study and sharing in a timely manner any new information that may be relevant to the patient's willingness to continue his or her participation in the trial.

4.1.2 General Guidelines for MD Anderson Cancer Center and Houston- Area Locations

Patients will be registered and randomized in central registration system by the research team in the Breast Radiation Oncology Section at the University of Texas MD Anderson Cancer Center or

by the staff within the Office of Protocol Support and Management for patients enrolled through the Houston Area Locations.

4.1.3 General Guidelines for Cancer Network Locations

Eligible participants will be registered and randomized centrally at MD Anderson Cancer Center by a member of the Research team prior to the initiation of treatment.

To register a participant, the following documents should be uploaded into the appropriate institutionally approved cloud-based storage folder:

- Signed informed consent form
- HIPAA authorization form (if separate from the informed consent document)
- Completed eligibility checklist with supporting source documents
- Pathology report of invasive breast cancer and stage of the tumor
- Clinic visit note documenting consent process, history and physical exam, cardiac risk factor assessments, treatment plan(s) and the planned date of radiation treatment

After the central registration and randomization process, an email confirmation of the registration, assigned protocol patient identification number (PPID#), and randomized group number will be sent to the participating Cancer Network Site by the assigned Cancer Network Research team member.

4.1.4 General Guidelines for Orlando Health

Eligible participants will be registered and randomized centrally at MD Anderson Cancer Center by a member of the Breast Radiation Oncology Section research team or by the Radiation Oncology multi-center coordinator prior to the initiation of treatment.

To register a participant, the following documents should be uploaded into an institutionally approved cloud-based storage folder (e.g., OneDrive):

- Signed informed consent form
- HIPAA authorization form (if separate from the informed consent document)
- Completed eligibility checklist with supporting source documents
- Pathology report of invasive breast cancer and stage of the tumor
- Clinic visit note documenting consent process, history and physical exam, cardiac risk factor assessments, treatment plan(s) and the planned date of radiation treatment

After the central registration and randomization process, an email confirmation of the registration, assigned unique subject identifier (SID), and randomized group number will be sent to Orlando Health by the assigned research team member and/or multi-center coordinator.

5 INVESTIGATIONAL PLAN

5.1 Study Design

5.1.1 This randomized trial will enroll up to 985 patients meeting the inclusion criteria outlined above to obtain up to 892 randomized patients. Patients will be recruited from MD Anderson Main Campus, Houston Area Locations, across the MD Anderson Cancer Network, and Orlando Health. The **Lymphedema Cohort** to evaluate arm lymphedema (N= up to 326 randomized participants) and the additional **Exploratory Cohort** of N=50 randomized participants who receive regional nodal irradiation before surgery are exclusively enrolled at the MD Anderson Cancer Center and the MD Anderson Cancer Center Houston- Area locations.

MD Anderson Cancer Center, MD Anderson Cancer Center Houston- Area locations, participating MD Anderson Cancer Network locations and Orlando Health will recruit up to 516 patients to the **Expanded Trial Cohort to evaluate locoregional control**.

5.1.2 Patients will be **randomized to** receive either the ***shorter*** **treatment (Group 1**), delivered over 3 weeks, or *the* ***standard of care*** **treatment (Group 2),** delivered over 5 weeks (see section 6 for dose specifications). Patients in both treatment arms will receive an additional 'boost' dose to the chest wall flaps (mastectomy patients) or tumor resection bed (segmental mastectomy patients). For patients receiving postoperative radiation therapy, randomization will be 1-1 and will be stratified by receipt of chemotherapy, extent of axillary evaluation, and patient body mass index as these factors contribute to the risk of treatment toxicity. For patients enrolling in the **Lymphedema Cohort**, randomization will also be stratified by pre-radiation perometry measurement. For patients receiving preoperative RNI, randomization will be 1:1 and will be stratified by pre-radiation perometry measurement and patient body mass index. Please refer to section 9.4 for detailed information about the stratification criteria.

The trial was initially designed to evaluate 290 patients with the primary endpoint of arm lymphedema. In 2018 the trial was amended to include a separate cohort of 30 patients who receive regional nodal irradiation before surgery. In the Fall of 2020, the trial was amended to enroll an additional up to 552 patients who will receive postoperative radiation (up to 842 postoperative patients total) to permit evaluation of locoregional cancer control. In October 2020 the protocol was amended to permit enrollment of up to 960 patients to obtain up to 872 *randomized* patients including up to 290 *randomized* patients for the lymphedema cohort, up to 842 *randomized* patients for the locoregional control cohort (up to 290 *randomized* patients from the lymphedema cohort plus up to 552 randomized patients from the expansion cohort to assess locoregional control) and 30 *randomized* patients for the preoperative cohort. In June 2021 the protocol was amended to increase the number of patients in the separate cohort of patients who receive regional nodal irradiation before surgery to 50 to provide more robust pilot data to inform future prospective trial design and to increase the number of patients in the lymphedema cohort to up to 326 due to higher than anticipated missed patient arm assessments during the COVID pandemic. Since the lymphedema cohort patients inform both the lymphedema and local control outcomes, the protocol now permitted enrollment of up to 985 patients to obtain up to 892 *randomized* patients including up to 326 *randomized* patients for the lymphedema cohort, up to 842 *randomized* patients for the locoregional control cohort (up to 326 *randomized* patients from the lymphedema cohort plus up to 516 *randomized* patients from the expansion cohort to assess locoregional control) and 50 *randomized* patients for the preoperative cohort. For Lymphedema cohort receiving postoperative radiation, up to 326 patients will be randomized in order to obtain at least 260 randomized & evaluable patients. For the locoregional control cohort, up to 842 patients (up to 326 *randomized* patients from the lymphedema cohort plus up to 516 *randomized* patients from the expansion cohort to assess locoregional control) will be randomized in order to obtain at least 716 randomized & evaluable patients.

5.1.3

5.1.4 Cancer control, physician-assessed skin and soft tissue toxicity, and patient-reported outcomes will be evaluated through 10 years after RNI. The burden of treatment will be assessed using validated questionnaires that assess local-regional symptoms and quality of life. For those patients

participating in the arm lymphedema cohort, the difference in arm volume assessed via perometry will be evaluated through 10 years after RNI. Peripheral blood will be stored for future biomarker analysis.

6 RADIATION TREATMENT/DOSE SPECIFICATIONS

6.1 Treatment target and prescription

6.1.1 External beam photon and/or electron radiation therapy will be used exclusively in this study. Brachytherapy and proton therapy are not allowed.

6.1.2 For patients receiving postoperative radiation therapy, radiation therapy must begin within 24 weeks of the later of these two dates: the date of final breast cancer surgery or the date of the last infusion of cytotoxic chemotherapy.

6.1.3 For patients undergoing mastectomy and receiving postoperative radiation therapy, the treatment target will be the chest wall and undissected lymphatics. For patients undergoing breast conserving surgery or preoperative radiation therapy, the treatment target will be the breast and undissected lymphatics. The supraclavicular and infraclavicular lymph nodes are part of the treatment target. The level I and II axilla are included if they were not surgically removed (a targeted axillary dissection may be considered surgical removal of the axillary nodes for the purposes of this protocol). Treatment of the internal mammary nodes is strongly encouraged but will be left to the discretion of the treating physician.

6.1.4 The prescription dose for patients assigned to the shorter RNI arm will be 40 Gy in 15 fractions to the breast or chest wall and 37.5 Gy in 15 fractions to the undissected lymphatics. Radiation will be delivered on consecutive treatment days. A treatment day is defined as a normal business day, typically Monday – Friday excluding institutional holidays.

6.1.5 The prescription dose for patients assigned to the standard RNI arm will be 50 Gy in 25 fractions to the breast or chest wall and 45 Gy in 25 fractions to the undissected lymphatics. Radiation will be delivered on consecutive treatment days. A treatment day is defined as a normal business day, typically Monday – Friday excluding institutional holidays.

6.1.6 Patients in both treatment arms who receive postoperative radiation will receive additional 'boost' dose to either the chest wall flaps (mastectomy patients) or tumor resection bed (segmental mastectomy patients). The prescription dose for boost will be 10 Gy in 5 fractions delivered on

consecutive treatment days for patients with negative margins (≥ 2 mm from DCIS or invasive carcinoma to the closest inked surgical margin) and 14 Gy in 7 fractions delivered on consecutive treatment days for patients with close surgical margins (< 2 mm from DCIS or invasive carcinoma and closest inked surgical margin). The boost will begin on the treatment day following completion of RNI. The boost may be omitted if such omission is clinically indicated; if omitted, document the clinical indication on an appropriate source. Patients who receive preoperative radiotherapy will not have a chest-wall boost. Nodal boost (10-16 Gy in 5-8 fractions) is recommended for patients with N3a disease (preoperative and postoperative cohorts) or N3b disease (preoperative cohort).

6.1.7 The prescription points for treatment will be selected at the discretion of the treating radiation oncologist to balance target coverage versus dose homogeneity.

6.1.8 Inhomogeneity corrections will be used in dose calculations.

6.1.9 Radiation treatment breaks due to unexpected toxicity are permitted but generally discouraged.

6.2 Technical Factors

6.2.1 Radiation will be delivered using a linear accelerator with a nominal energy ≥ 6 MV.

6.2.2 The treating physician may choose the best external beam radiation technique for covering the target volume. It is expected that forward planned, three-dimensional dose compensation will be the most commonly used technique.

6.2.3 Respiratory gating with the deep inspiration breath hold technique may be utilized to minimize exposure of the heart and/or lung to radiation when thought to be indicated by the treating radiation oncologist.

6.3 Localization, Simulation, and Immobilization

6.3.1 Patients will be immobilized using a breast board and vacuum-lock bag or other institution-specific immobilization techniques in standard use at the discretion of their treating physician.

6.3.2 Patients will undergo computed tomography-based simulation with an axial slice thickness no greater than 5 mm.

6.4 Treatment Planning/Target Volumes

6.4.1 All treatment plans at MD Anderson Cancer Center should adhere to Institutional best practices in place at the time of simulation and should be reviewed prospectively at Departmental quality assurance chart rounds as per standard practice.

6.4.2 For patients enrolled at participating MD Anderson Cancer Network locations, or Orlando Health, the radiation treatment plans for the first 5 patients will be required to undergo a central review by Dr. Karen Hoffman and/or Dr. Benjamin Smith prior to beginning protocol- specified radiotherapy. Cancer Network Sites and Orlando Health are required to upload the radiation therapy treatment plans into an institutionally-approved cloud-based storage folder including the planned dates of radiation therapy. Generally, these treatment plans will be reviewed and approved within 24 hours by the MD Anderson team.

6.4.3 For patients undergoing mastectomy, the mastectomy scar and chest wall should be delineated clinically using radiopaque wire at the time of simulation. For patients undergoing breast conserving surgery the lumpectomy scar and breast mound or clinical treatment borders should be delineated at the time of simulation.

6.4.4 For all patients, the undissected lymphatics target should be contoured on the CT scan obtained at simulation. This will include the level III medial infraclavicular and supraclavicular lymph node basins. For patients receiving treatment to the internal mammary lymph nodes, this target should also be contoured on the CT scan obtained at simulation. Typically, this will include the first three interspaces, from the caudal edge of the first rib to the cranial edge of the fourth rib. For patients receiving treatment to the level I and II axilla, this structure should also be contoured on the CT scan obtained at simulation.

6.4.5 The treating physician should aim to cover the chest wall or breast to ensure that the 98% isodose line covers the clinical target without breaking up. An exception to this guideline would be patients treated with electron chest wall plans, where acceptable coverage ensures that the 90% isodose line does not break up.

6.4.6 The treating physician should aim to cover the contoured regional lymph node targets to ensure that the minimum dose to these structures is the prescription dose (i.e. minimum coverage by the 100% isodose line). If only greater than 95% of the contoured regional lymph nodes is covered by the 100% isodose line, this will be considered an acceptable variation.

6.5 Critical Structures

6.5.1 For patients treated with standard RNI, the ipsilateral lung V20 should ideally be less than 35%. Less than 45% is acceptable. The mean heart dose should ideally be less than 4 Gy. Less than 6 Gy is

acceptable. The maximum dose to the spinal cord should be less than 45 Gy, but this structure need not be contoured if it is clearly outside the 36 Gy isodose line.

6.5.2 For patients treated with shorter RNI, the ipsilateral lung V16 should ideally be less than 35%. Less than 45% is acceptable. The mean heart dose should ideally be less than 3.2 Gy. Less than 4.8 Gy is acceptable. The maximum dose to the spinal cord should be less than 36 Gy, and this structure need not be contoured if it is clearly outside the 30 Gy isodose line.

6.5.3 These dose constraints do not include the boost doses. The boosts should seek to deliver normal tissue doses as low as reasonably achievable.

6.6 Compliance Criteria

6.6.1 Treatment plans will be considered acceptable if they adhere to the treatment targets and dose constraints outlined in Sections 6.4 and 6.5.

6.6.2 A treatment break of up to 3 treatment days is acceptable. A break of 4 or more days will be considered a variation and must be reported to lead PI via an appropriate form.

6.6.3 If the patient receives less than 95% of the intended radiation dose but more than 90% of the intended radiation dose this will be considered a minor violation. If the patient receives less than 90% of the intended radiation dose this will be a major violation. If a patient receives less dose of radiation because he or she does not comply with radiation, this shall not be considered a violation and the reason(s) must be documented on an appropriate source. If the boost is omitted based on clinical grounds, this will not be considered a violation or variation and the reason(s) must be documented on an appropriate source as well. The deviation or violation forms created specifically for MD Anderson Cancer Center will be provided to each site and sites are required to complete this form(s) and upload it into an institutionally approved cloud-based storage folder in a timely manner if any violation/deviation occurs.

6.6.4 If the patient has a COVID-19 exposure or positive COVID-19 test that results in a treatment break, treatment delay, change in total radiation dose or change in dose per fraction it will be considered a variation and must be reported to lead PI via an appropriate form. Source documentation will need to be provided for either a positive COVID-19 test or COVID-19 exposure if any of the above changes occur from the participant's original protocol- specified radiation therapy regimen.

6.7 Radiation Therapy Quality Assurance Reviews

6.7.1 All cases will be presented for peer review in accordance with the policy of the Department of Radiation Oncology at The University of Texas MD Anderson Cancer Center. For patients enrolled at participating MD Anderson Cancer Network locations, or Orlando Health, the site's PI is responsible to monitor the progress of the protocol and safety of the participants in accordance with the protocol, and all applicable privacy laws, rules, and regulations.

6.8 Radiation Adverse Events

6.8.1 Events that may occur include fatigue, skin erythema, dry or moist desquamation, hyperpigmentation, alopecia, tenderness, and swelling. Uncommon side effects include cellulitis or severe breast pain. In the long term, breast and chest wall changes may occur including hyper- or hypo-pigmentation, telangiectasia, fibrosis and lymphedema. Uncommon side effects that may occur after completion of radiation include rib fracture, pneumonitis, pulmonary fibrosis, pericarditis, brachial plexus injury, ischemic heart disease, and heart failure.

6.9 Adverse Events and Serious Adverse Events Reporting Requirements

6.9.1 Adverse events will be described and graded using the terminology and grading categories of the NCI's Common Toxicity Criteria (CTCAE) version 4.0. For toxicities that do not have a recommended system grading criterion under the CTCAE and do not fit into an existing category, use "Other" and specify toxicity (i.e. Metabolic/Laboratory – Other, Elevated BUN). The treating physician and investigator team are responsible for the detection, documentation, and reporting of events meeting the criteria and definition of an adverse event (AE) or serious adverse event (SAE). As radiation therapy is a standard treatment, neither date of onset, date of cessation, nor attribution will be collected.

6.9.2 Adverse events both nonserious and serious, will be monitored throughout the study as follows:

- Adverse events will be reported from the first dose of radiation through 42 days after administration of the last dose of radiation therapy and recorded in the appropriate eCRFs and source document.
- All serious adverse events that occur after the study initiation, during treatment, or within 42 days of the last dose of radiation treatment must be reported to the MD Anderson Lead Principal Investigator on the appropriate SAE form within 24 business hours of learning of

the occurrence. Follow-up information on the SAE event may be requested by MD Anderson Cancer Center. The presence and resolution of SAEs (with dates) should be documented on the appropriate case report form and recorded in the participant's medical record or source to facilitate source data verification. The SAE Form, created specifically by MD Anderson Cancer Center, will be provided to each participating Cancer Network Site and Orlando Health. A sample of the SAE Form may be found in the institutionally approved cloud-based storage folder as well.

- Participating Cancer Network Sites and Orlando Health should report SAEs to their respective IRB according to the local IRB's policies and procedures and a copy of the submitted institutional SAE form should be uploaded into the appropriate institutionally approved cloud-based storage folder.

7 PERMITTED SUPPPORTIVE THERAPY

7.1.1 All supportive therapy indicated for optimal medical therapy during the course of radiation will be permitted for this study. Supportive therapy may include, but is not limited to, the following:

1) Radiodermatitis creams or ointments
2) Topical steroids
3) Oral antipruritics
4) Oral analgesics to include non-narcotic and narcotic medications
5) Antibiotic therapy as indicated for infection
6) Nutritional supplementation

8 EVALUATION DURING STUDY

8.1 Physician visits

8.1.1 During treatment. Patients will undergo weekly evaluations during radiation therapy as per standard of care. These will be documented in the medical record. Participating Cancer Network Sites and Orlando Health may provide a weekly evaluation report (uploaded into an appropriate cloud-based folder) if requested by MD Anderson Cancer Center. At the completion of treatment, the treating physician will record all acute toxicities experienced during radiation. These will be

documented either on the radiation treatment summary in the medical record or on a separate form (Follow Up and Adverse Events) signed by the treating physician. The type and grade of each toxic event experienced during radiation treatment will be recorded. Investigators can complete the Follow up and Adverse Event form through REDCap. Participating Cancer Network Sites and Orlando Health are required to upload the radiation treatment summary document into the appropriate institutionally approved cloud-based folder. Additional toxic events experienced within 42 days of completion of radiation will also be considered acute toxicities in all analyses. As radiation therapy is a standard treatment, neither date of onset, date of cessation, nor attribution will be collected.

8.1.2 After completing radiation treatment. Patients will return for physician evaluations 6 months after completing radiation (+/4 months) and yearly thereafter (+/- 6 months) through 126 months. At each physician evaluation, disease status and the type and grade of each toxic event will be recorded by using the Follow Up and Adverse Event form. Investigators can complete the Follow up and Adverse Event form through REDCap. Additional physician visits that occur between 43 days after completion of radiation and the end of the study may also be recorded for type and grade of toxic events. As radiation therapy is a standard treatment, neither date of onset, date of cessation, nor attribution will be collected. Any toxic event documented between 43 days after completion of radiation and the completion of all study-related follow up will be considered a late event in all analyses.

8.1.3 After completing 10.5 years of follow up, patients will be off study but may continue to be followed by the attending radiation oncologist or discharged to either the Institution's Cancer Survivors Clinic, if available, or to their primary medical doctor.

8.1.4 If study subjects are unable to see a physician in follow up due to loss of insurance coverage, toxicity and disease control can be assessed by the designated research staff by using institutional resources. If patient is unable to return to the treatment center for follow up, research staff may request patient permission to obtain outside medical records to review for disease control and toxicity. In the instance that a participant is unable to be contacted or seen in clinic to collect their protocol- required survey(s) or assessments in long term follow-up, the missing/ not collected data will not be considered a protocol deviation. For the purposes of this research protocol, long term follow-up is defined as greater than or equal to 5- years post- radiation therapy.

8.2 Surveillance imaging

8.2.1 Surveillance imaging assessment during follow up should adhere to best medical practice, which typically includes annual mammography for patients with intact breast(s). Other studies such as ultrasound or cross-sectional imaging may be obtained but are not required. Participating Cancer Network Sites and Orlando Health are required to submit imaging reports into an institutionally approved cloud-based folder. The schedule for surveillance mammography will be at the discretion of the treating physician.

8.3 Other research-related assessments

8.3.1 Volume measurements (not applicable for Cancer Network Site and Orlando Health patients). For patients enrolled in the arm lymphedema cohort, volume measurements of both arms will be obtained using a perometer prior to radiation and at the 3, 6, 12, 18, 24, 66, and 126-month timepoints. Perometers quantitate arm volume by infrared technology and assessments have high intrarater (0.997) and interrater (0.997) reliability (Deltombe 2007). Perometer-based arm measurement of women under treatment for breast cancer is a standard of care evaluation in the breast center. The breast center strives to capture all patients with invasive breast cancer preoperatively and follow those at high risk of developing lymphedema as part of standard of care. There are two Breast Center Lymphedema Medical Assistants who perform perometer-based arm measurements in the breast center. Perometers are also used to assess patients for lymphedema in the physical therapy department. The perometers are manufactured by Messgërate GmbH. The perometers are maintained according to the manual which recommends visual inspection and test of functions twice per year and replacement of the balancer every five years. The device does not have FDA clearance, but it is commonly used to measure arm volume, is a standard of care measurement in our breast center, and there is extensive literature to support the use of perometery devices. Patients found to have a 10 or greater relative percent difference in arm volume at time of perometer measurement, may be referred to physical therapy for evaluation and treatment if they have not previously received lymphedema therapy. This threshold shall be used to define the presence of clinical lymphedema at the measurement timepoint.

8.3.2 EuroQOL EQ-5D (Pickard 2007). The EQ-5D-3L will be used to measure patient-reported health related quality of life and yields a utilities index value for health status. The first part of the instrument has five questions covering five dimensions (mobility, self-care, usual activities, pain/discomfort, anxiety/depression) and classifies respondents into distinct health states. The

second part is a visual analog scale valuing current health state with worst imaginable health state scored as 0 at the bottom of the scale, and the best imaginable health state scored as a 100 at the top. The EQ-5D-3L will be administered prior to radiation and at the 3, 6, 12, 18, 24, 66, and 126-month timepoints. The EQ-5D-3L will be administered in English and Spanish and is not required for patients who are not fluent in either language. Patients will have the option to complete this form on paper, or through electronic mode (REDCap). Patients may also be contacted by phone to complete the form. Participating Cancer Network Sites and Orlando Health are required to upload the collected EQ-5D-3L into an institutionally approved cloud-based folder if collected in a paper format.

8.3.3 The Disabilities of Arm, Shoulder and Hand (QuickDASH—9, Gabel 2009). The QuickDASH—9 is a validated 9-item questionnaire that evaluates arm and shoulder symptoms and the ability to perform certain tasks. . The QuickDASH-9 will be administered prior to radiation and at the 3, 6, 12, 18, 24, 66, and 126- month timepoints. The QuickDASH-9 will be administered in English and Spanish and is not required for patients who are not fluent in either language. Patients will have the option to complete this form on paper, or through electronic mode (REDCap). Patients may also be contacted by phone to complete the form. Participating Cancer Network Sites and Orlando Health are required to upload the collected QuickDASH - 9 into an institutionally approved cloud-based folder if collected in a paper format.

8.3.4 PROMIS Fatigue Short Form (PROMIS Fatigue SF 6a) (Cella 2010; Garcia 2008). The six-item PROMIS Fatigue short form evaluates self-reported symptoms of fatigue over the past seven days. It assesses both the extent of fatigue as well as the impact of fatigue on activities. It will be administered prior to radiation and at the 3, 6, 12, 18, 24, 66, and 126-month timepoints. It will be administered in English and Spanish and is not required for patients who are not fluent in either language. Patients will have the option to complete this form on paper, or through electronic mode (REDCap). Patients may also be contacted by phone to complete the form. Participating Cancer Network Sites and Orlando Health are required to upload the collected PROMIS Fatigue SF 6a into an institutionally approved cloud-based folder if collected in a paper format.

8.3.5 Satisfaction with Cosmetic Outcome Questionnaire (Jagsi 2015). This six-item scale was developed to provide a brief assessment of patient-reported cosmetic outcomes after breast cancer treatment. It will be administered prior to radiation and at the 3, 6, 12, 18, 24, 66, and 126- month

timepoints. It will be administered in English and Spanish and is not required for patients who are not fluent in either language. These questions are optional for men enrolled in the trial. Patients will have the option to complete this form on paper, or through electronic mode (REDCap). Patients may also be contacted by phone to complete the form. Participating Cancer Network Sites and Orlando Health are required to upload the collected Satisfaction with Cosmetic Outcome Questionnaire into an institutionally approved cloud-based folder if collected in a paper format.

8.3.6 Additional questions regarding arm function will also be administered prior to radiation and at the 3, 6, 12, 18, 24, 66, and 126-month timepoints. These questions were obtained from the Patient-reported arm function questions (Arm Function FACT B+4) and evaluate pain or arm movement, arm range of motion, arm numbness and arm stiffness. It will be administered in English and Spanish and is not required for patients who are not fluent in either language. Patients will have the option to complete this form on paper, or through electronic mode (REDCap). Patients may also be contacted by phone to complete the form. Participating Cancer Network Sites and Orlando Health are required to upload the collected information source into an institutionally approved cloud-based folder.

8.3.7 Assessment of cardiac risk factors. Additional questions will be asked about the presence or absence of cardiovascular risk factors including history of coronary artery disease or myocardial infarction, atrial fibrillation/flutter, diabetes, hypertension, renal failure, hyperlipidemia, heart failure, cardiomyopathy, and smoking history (current/former/never) prior to radiation and at the 6, 18, 30, 42, 54, 66, 78, 90, 102, 114, and 126-month timepoints. We will record blood pressure and family history of cardiovascular disease prior to the radiation timepoint. This assessment will permit us to calculate Framingham risk scores for development of cardiovascular disease for patients (D'Agostino 2008) and will provide additional detail about cardiovascular risk factors. Participating Cancer Network Sites and Orlando Health are required to upload the collected information source into an institutionally approved cloud-based folder.

8.3.8 Echocardiography with GLS measurements. Patients may consent for cardiac strain evaluation by non-invasive echocardiograms. This endpoint is optional. It is not applicable for participating Cancer Network Sites or Orlando Health. Echocardiograms will be obtained prior to radiation, the final week of radiation and at the 6, and 12 month timepoints. Echocardiograms will be performed by experienced sonographers using a GE machine (Vivid 7;

GE Healthcare, Milwaukee, WI, USA) with a 2.5 or 3.5 MHz phased array transducer. 2D and color Doppler images from the apical four-, three- and two-chamber views will be acquired, and all echocardiographic measurements will be performed offline in a blinded fashion. Quantitative LVEF will be measured using the Bi-plane Simpson's Method. Diastolic parameters including early mitral inflow velocity (E), the late diastolic velocity (A), the deceleration time and, the E/A ratio, the systolic velocity of the medial mitral annulus (Smed), the early diastolic velocity of the medial mitral annulus (E'med), the late diastolic velocity of the medial mitral annulus (A'med), the systolic velocity of the lateral mitral annulus (Slat), the early diastolic velocity of the lateral mitral annulus (E'lat), and the late diastolic velocity of the lateral mitral annulus (A'lat) will be measured. Global longitudinal strain will be measured using 2D speckle-tracking echocardiography with 3 long axis images obtained at high frame rates (50-70fps). Analysis will be performed using the EchoPAC software (version BT11, GE Medical, Milwaukee, WI, USA).

8.3.9 ..... Assessment of surgical complications. Standard documentation of any intraoperative difficulties noted by the surgical oncologist and plastic surgeon at surgeries performed after radiation will be noted from the operative reports. Evaluation of surgical and reconstructive complications will include: time between surgery and radiation; if surgery had to be delayed due to radiation skin or soft tissue effects; abortion of reconstructive procedure due to radiation skin, soft tissue, or recipient vasculature effects; mastectomy skin flap necrosis requiring reoperation or debridement; complete or partial autologous flap loss; surgical recipient site delayed wound healing complication; surgical recipient site infection requiring oral or intravenous antimicrobial therapy; and loss of implant or tissue expander secondary to extrusion or infection.

8.3.10 Timing of research-related assessments. Assessments scheduled to coincide with physician visits will be conducted at this timepoint whenever feasible. Assessments scheduled for 3, 12, and 24 months after completion of radiation do not correspond with a physician visit. For assessments due at these timepoints, timing is intended to be flexible. The 3 month visit should occur at any time between end of treatment and the 6 month physician visit. Similarly, the 12 month assessment should occur at any time between the 6 month physician visit and the 18 month physician visit, and the 24 month assessment should occur at any time between the 18 month physician visit and the 36

month physician visit. Time windows for all assessments scheduled to coincide with physician assessments will adhere to the time windows scheduled for physician assessments.

8.3.11 The ENRICh Instrument (also known as the Economic Strain and Resilience in Cancer- Financial Well- Being instrument)This questionnaire evaluates self-reported severity of financial burden from having cancer and cancer treatment (Smith G 2019). It will be administered prior to radiation, final week of treatment, and at the 3, 6, 12, 18, 24 and 66-month timepoints. Patients will have the option to complete this form on paper, or through electronic mode (REDCap). Patients may also be contacted by phone to complete the form. Participating Cancer Network Sites and Orlando Health are required to upload the collected Economic Strain and Resilience in Cancer- Financial Well- Being instrument into an institutionally approved cloud-based folder if collected in a paper format.

8.3.12 Photographic documentation of cosmesis. For patients in the preoperative radiation cohort only, photographs will be taken prior to radiation, the final week of treatment, and at the 3, 6, 12, 18, 24 and 66 month timepoints. Photographs will be framed to include the low neck down to the upper abdomen and will be taken with arms at the side and patients will be asked to remove all jewelry prior to photography. Five views will be obtained: right lateral, right anterior oblique, anteroposterior, left anterior oblique, and left lateral. All photographic data will be stored on a password-protected computer.

8.4 Blood procurement and analysis (designed **only** for UT MD Anderson Cancer Center and Houston Area Locations' patients)

8.4.1 Patients may consent to optional blood draw within 3 months prior to starting radiation (or within the first week of starting), for fasting lipid profile (HDL, LDL, total cholesterol, triglycerides) and homocysteine measured from peripheral blood using standard of care laboratory protocols.

8.4.2 Patients may also consent to cytokine collection from peripheral blood, which is optional. Cytokines shall be collected prior to radiation and during the last week of radiation. A third cytokine collection specimen may be obtained at the first time that the patient is found to have developed lymphedema (10% or greater standardized difference in extremity volume) based on perometry measurement. To collect cytokines, one red-stopper vacutainer tube will be used. Custom multi-plex cytokine profiles in peripheral blood will be

examined using luminex 96 well plates. Post treatment samples will be normalized by pretreatment controls.

8.4.3 Patients may also consent to a peripheral blood collection to evaluate changes in double strand break repair and extraction of DNA, which is optional. Blood will be drawn at each of three time points during the course of the trial: prior to radiation, during the last week of radiation, and at the three month follow up visit. To collect blood for these assays, one to two tubes of blood will be collected using a mononuclear cell preparation (CPT) tube and/or a sodium heparin (green top) tube. Blood will initially be stored and processed in Dr. Wendy Woodward's laboratory in the Department of Radiation Oncology. After a material transfer agreement is obtained, the blood will be transported to Dr. El-Zein's core laboratory at Houston Methodist Research Institute for processing and performing the assays as outlined in the material transfer agreement. The specimen will be transported without patient identifiers except for a unique ID that can be traced back to the master spreadsheet kept in a password protected institutional computer. The data is not stored in the hard drive but maintained in an institutional server that is backed up and password secured. Only the PI and research team at the respective institutions have access to this file.

8.4.4 Patients may also consent to a peripheral blood collection to conduct DNA sequencing of peripheral blood cells to examine for the presence and characteristics of clonal hematopoiesis mutations, which is optional. Blood will be collected prior to radiation, the final week of radiation, and 6 and 12 months after radiation. One to two tubes of whole blood (EDTA additive, "lavender" top tube) will be collected at each timepoint. Exome peripheral blood DNA sequencing will be conducted at MD Anderson Cancer Center on each sample.

8.4.5 Patients may also consent to a peripheral blood collection to evaluate cardiac biomarkers, which is optional. Blood will be collected prior to radiation, the final week of radiation and 6 and 12 months after radiation. One to two tubes of serum (no additive, "red" top tube) and one to two tubes of whole blood (citrate additive, "blue" top mononuclear cell preparation tube, CPT) will be collected at each timepoint. Commercially-available enzyme-linked immunosorbent assays (ELISA) will be performed for cardiac biomarkers including:

high sensitivity troponin, BNP, NT-proBNP, galectin-3, MPO, high sensitivity CRP, PIGF, NO, GDF-15, H-FABP, GPBB, sFlt—1 and TGF-beta.

8.4.6 Patients may consent for leftover blood from optional testing will be stored in a research tissue bank at MD Anderson for use in future research. As new protocols are developed to use this blood they will be presented for IRB review; if approval is obtained, the stored samples may be used for additional research projects.

8.4.7 These optional blood draws can be performed MD Anderson diagnostic laboratories. Blood will then be transferred to Dr. Woodward's laboratory for initial processing and storage according to standard operating procedures. All specimens will be entered in tissue station as per institutional requirements.

8.4.8 -- Schedule of Events

MD Anderson Cancer Center and Houston- Area Locations

	**Timing of Patient Evaluations**																
				**Months post completion of radiation**													
Assessments / Procedures	Prior to RT	Weekly during RT	Final week RT*	3#	6†	12 ##	18 †	24 ###	30 †	42 ‡	54 ‡	66‡ %	78‡ %	90‡ %	102‡ %	114‡ %	126‡ %
Physical exam, disease, and treatment histories	X																
Blood pressure and family history of cardiovascular disease	X																
Assessment of cardiac risk factors including smoking history	X				X		X		X	X	X	X	X	X	X	X	X
Perometry (lymphedema cohort)	X			X	X	X	X	X				X					X
Patient-reported arm function questions (Arm Function Fact B+4)	X			X	X	X	X	X				X					X
QuickDASH-9	X			X	X	X	X	X				X					X
EQ-5D-3L	X			X	X	X	X	X				X					X
Satisfaction with cosmetic outcome questions	X			X	X	X	X	X				X					X
PROMIS Fatigue SF 6a	X			X	X	X	X	X				X					X
Economic Strain and Resilience in Cancer- Financial Well- Being instrument	X		X	X	X	X	X	X				X					
Photographic assessment (preop cohort)	X		X	X	X	X	X	X				X					
Acute toxicity assessment			X														
Dosimetry recorded			X														
Physician visits (Follow Up and Adverse Events)			X		X		X		X	X	X	X	X	X	X	X	X
Blood draw lipids & homocysteine^	X																
Blood draw cytokines^	X		X	X**	X**	X**	X**	X**				X**					X**
Blood draw DNA repair^	X		X	X													
Blood draw clonal hematopoiesis^	X		X		X	X											
Blood draw cardiac biomarkers^	X		X		X	X											
Cardiac strain echo^	X		X		X	X											

Lymph nodes for immune system testing^	X																
Surveillance image(s)@							X		X	X	X	X	X	X	X	X	X

* To be completed within 2 weeks of completing radiation

Occurs any time between completion of radiation and 6 months after radiation

† After end of radiation +/- 4 months

Occurs any time between 6-month physician assessment and 18-month physician assessment

‡ After end of radiation +/- 6 months

Occurs any time between 18-month physician assessment and 30-month physician assessment

**Blood may be collected at the first perometry measurement after radiation at which lymphedema (?10% standardized difference in extremity volume) is diagnosed.

^Optional blood draw or procedure only designed for MD Anderson Cancer Center or Houston Area Locations patients

@At physician discretion and/ or as per standard of care (SoC)

% In the instance that a participant is unable to be contacted or seen in clinic to collect their protocol- required survey(s) or assessments in long term follow-up, the missing/ not collected data will not be considered a protocol deviation.

Abbreviations: QuickDASH-9=Disabilities of Arm, Shoulder and Hand; RT (radiation therapy)
Rev09172021

8.4.9 -- Schedule of Events

MD Anderson Cancer Network Sites or Orlando Health

	Timing of Patient Evaluations																
				Months post completion of radiation													
Assessments / Procedures	Prior to RT	Weekly during RT*	Final week RT**	3#	6†	12 ##	18†	24 ###	30†	42‡	54‡	66‡%	78‡ %	90‡ %	102‡ %	114‡ %	126‡ %
Physical exam, disease, and treatment histories	X																
Blood pressure and family history of cardiovascular disease	X																
Assessment of cardiac risk factors including smoking history	X				X		X		X	X	X	X	X	X	X	X	X
Patient-reported arm function questions (Arm Function Fact B+4)	X			X	X	X	X	X				X					X
QuickDASH-9	X			X	X	X	X	X				X					X
EQ-5D-3L	X			X	X	X	X	X				X					X
Satisfaction with cosmetic outcome questions	X			X	X	X	X	X				X					X
PROMIS Fatigue SF 6a	X			X	X	X	X	X				X					X
Economic Strain and Resilience in Cancer- Financial Well- Being instrument	X		X	X	X	X	X	X				X					
Acute toxicity assessment			X														
Dosimetry recorded			X														
Physician visits (Follow Up and Adverse Events)		X	X		X		X		X	X	X	X	X	X	X	X	X
Surveillance image(s)[@]							X		X	X	X	X	X	X	X	X	X

* Weekly evaluations during radiation therapy as per the standard of care and CN Sites and Orlando Health may provide a weekly evaluation report to MD Anderson Cancer Center if requested. Weekly evaluation data is not required to be captured in REDCap.

** To be completed within 2 weeks of completing radiation

Occurs any time between completion of radiation and 6 months after radiation

† After end of radiation +/- 4 months

Occurs any time between 6-month physician assessment and 18-month physician assessment

‡ After end of radiation +/- 6 months

Occurs any time between 18-month physician assessment and 30-month physician assessment

@At physician discretion and/ or as per standard of care (SoC)

% In the instance that a participant is unable to be contacted or seen in clinic to collect their protocol- required survey(s) or assessments in long term follow-up, the missing/ not collected data will not be considered a protocol deviation.

Abbreviations: QuickDASH-9=Disabilities of Arm, Shoulder and Hand; RT (radiation therapy)

Rev09172021

9 STATISTICAL CONSIDERATIONS

9.1 Sample size calculation for locoregional control.

To evaluate the impact of shorter RNI on 5-year locoregional recurrence (LRR), a total of up to 842 randomized patients receiving postoperative radiation will be enrolled in the study. An evaluable patient will be considered a patient who is confirmed to meet all eligibility criteria, randomized, and undergoes either standard RNI or shorter RNI.

We assume that at 5 years, 97% patients would be alive and locoregional recurrent free, and we also assume a non-inferiority margin of 3% (corresponding to a null HR = 2 and alternative HR = 1). Given an accrual rate of 40 patients in the first year, 100 patients/year for the second and third year, 50 in the fourth year, and assuming an accrual rate 150 patients/year starting at 4th year, with additional 8 years of follow-up, and total dropout rate of 15%. A total of 716 patients will yield 80% power at a 1-sided 0.05 significance level. The maximum number of events that need to be observed is 39. We plan to have one interim analysis for futility when half of our expected observed events occurred. A Lan and DeMets spending function with O'Brien-Fleming boundary will be used. The trial will be stopped early if the hazard ratio is greater than or equal to 1.962 at the interim analysis. East 6.5 was used for sample size calculation.

9.2 Sample size calculation and analysis of lymphedema.

9.2.1 The primary objective for the arm lymphedema cohort is to compare the lymphedema rate during the RNI and 24 months after the completion of RNI between patients randomized to short versus standard RNI. All arm volume measurements will be obtained using a perometer, which provides a quantitative measurement of each arm's volume (Deltombe 2007) prior to surgery, post-operatively and then 5 times (3, 6, 12,18 and 24 months) over 24 months after completion of radiation. We define the standardized difference as the ratio of the volume measurements of the affected arm to the control arm, normalized for the presurgical baseline difference in arm volume,

$$\text{Standardized Difference} = \frac{ratio_{post} - ratio_{pre}}{ratio_{pre}},$$

where we will use the presurgical measurement obtained as a standard of care prior to surgery to determine the $ratio_{pre}$.

Lymphedema is defined as a >=10% standardized difference in arm volume. Any lymphedema on one of these 5 measurements after surgery is considered an event for that patient. With 260 evaluable patients with perometer measurement of lymphedema after RNI, 130 in each group, we will have an 82% power to detect a lymphedema rate of 15% in the short RNI group against 30% in the standard RNI group, using two-sided chi-squared test with a significance level of 0.05. Considering 20% attrition rate, we will need to randomize up to 326 patients, up to 163 in each group. In addition, we will enroll and randomize 50 patients who are offered mastectomy preoperative RNI by their physicians. The sample size was increased from 30 patients to 50 patients because of considerable interest in this pilot study and to provide more robust pilot data to inform future prospective trial design(s). The purpose is to collect and describe the occurrence of lymphedema in 2 years, the reconstructive complications, and explore any differences in these complications between patients with pre-surgery RNI and post-surgery RNI. However, if we observe at least 30% of the patients with reconstruction surgeries experience failed reconstruction after a total of 15 patients, we will halt the enrollment into this cohort.

9.2.2 An important secondary outcome measure to define lymphedema for this trial is the standardized difference between volume of the affected arm and the unaffected arm. At the end of the study, treating this measure as a continuous variable, the Shapiro-Wilk test will be used to objectively assess the normality of the data and these findings will be visually confirmed by inspecting normal probability Q-Q plots. If the data are not normally distributed, we may employ a normalizing transformation before using t-test or use a non-parametric method such as the Wilcoxon rank sum test to compare the standardized difference between the two arms at each time point. Due to the longitudinal nature of the measures of the volumes of arms, repeated measures analysis may also be employed to assess the effect of treatment over time on the ratio, adjusted for other important covariates.

9.3 Cohorts for primary analyses

9.3.1 For the **Lymphedema Cohort** to evaluate arm lymphedema, the primary analysis will be performed to compare risk of developing lymphedema based on the up to 326 patients enrolled in the cohort.

9.3.2 For the **Expanded Trial Cohort** to evaluate locoregional control, the primary analysis will be performed to compare risk of locoregional recurrence based on all up to 842 postoperative radiation patients.

The primary analysis will be conducted based on the intention to treat (ITT) approach where the population includes all subjects randomized to the two arms. The efficacy analysis will also be conducted based on the modified intent to treat patient populations: modified ITT population 1 and modified ITT population 2. Under modified ITT 1, the evaluable patients are defined as all subjects who meet all the eligibility criteria, randomized to the two arms and received the treatments. Under modified ITT 2, the evaluable patients are defined as all subjects who meet all the eligibility criteria, randomized to the two arms, get the assigned treatment, and has the primary endpoint information available.

9.4 Randomization and blinding

We will use the Pocock-Simon randomization method of dynamic allocation to balance the arms of the study for receipt of chemotherapy (none vs. neoadjuvant with or without adjuvant vs. adjuvant chemotherapy only), extent of axillary evaluation (axillary lymph node dissection vs. sentinel node evaluation), patient body mass index (less than 30 vs. 30 or greater), and presence of difference in arm volume on the post-operative, pre-radiation perometry measurement (less than 5% difference vs. 5% or greater difference).

For randomization and stratification, the patients in the **Lymphedema Cohort** to evaluate arm lymphedema receiving postoperative radiation, will be stratified by all four factors (receipt of chemotherapy, extent of axillary evaluation, patient body mass index and arm volume difference). The patients in the **Expanded Trial Cohort** to evaluate locoregional control receiving postoperative radiation will be stratified by three factors (receipt of chemotherapy, extent of axillary evaluation, and patient body mass index). For patients receiving preoperative RNI, randomization will be 1:1 and will be stratified by pre-radiation perometry measurement (less than 5% difference vs. 5% or greater difference) and patient body mass index (less than 30 vs. 30 or greater) only. The randomization will be 1:1 and will happen after the surgery for patients receiving postoperative

radiation therapy. It is not possible to blind patients and physicians to the number of radiation treatments a patient receives. However, the primary outcome is based on infrared measurement of arm volume by perometer and this automated measurement of arm volume cannot be influenced by physician or patient perception of the expected outcome.

All eligible patients will be required to provide informed signed consent before randomization and will be registered in OnCore. Randomization will be performed using the Clinical Trial Conduct website (https://biostatistics.mdanderson.org/ClinicalTrialConduct), which is maintained by the Department of Biostatistics. Research personnel responsible for enrolling and randomizing patients on this trial will be trained by the study statistician in the use of the website. Access to the website is gained through usernames and passwords. The sample size modification will be made accordingly for the CTC website.

9.5 Stopping rule

Since the 5-year locoregional recurrence (LRR) rate is about 3% for the standard RNI, the chance to observe an LR within the enrollment period is low. Therefore, besides the annual MDACC DSMB monitoring of the trial, the efficacy monitoring will be limited to the times when any LR is observed in the short RNI group before the last patient enrollment. At that time, we will estimate and compare LRR using time to event analysis between the two groups if appropriate and report to MDACC DSMB. Cumulative incidence function may be considered if non-ignorable number of patients die without locoregional recurrence or distant metastasis. The accrual will not be halted when this happens, however the occurrence of LR will be monitored closely and study statistician will be notified immediately to analyze the data, for both the pre-operative and post-operative radiation groups, when LR is observed. Decision to continue the trial (or not) will be made at that time by MDACC DSMB.

9.6 Patient-reported outcomes and Physician-assessed toxicity

Patient reported outcomes, including financial burden (assessed using the Economic Strain and Resilience in Cancer- Financial Well- Being instrument) physician-assessed acute toxicity, and physician-assessed late toxicity will be compared between patients treated with short versus standard RNI using chi-square statistic or Fisher’s exact test, t-test or Wilcoxon rank sum test, as appropriate, depending on the distribution of the outcomes.

9.7 Analysis of time to locoregional recurrence, time to distant metastasis, disease-free survival and overall survival

Patient demographic/clinic-pathological characteristics will be summarized at the end of the study using frequency tabulation for categorical variables or descriptive statistics (mean, standard deviation, median, and range) for continuous variables. Comparisons of these variables between treatment groups may be made by chi-squared test/Fisher's exact test or Wilcoxon rank sum test, when appropriate.

Time to locoregional recurrence (TLRR), time to distant metastasis (TDM), disease-free survival (DFS) time and overall survival (OS) time will be calculated from the randomization date to event date or last follow-up date. When analyzing TLLR and TDM, we may use the cumulative incidence function because the usual Kaplan–Meier survival-based estimates may be biased due to patients dying without locoregional recurrence or distant metastasis being counted as simple censoring events rather than as competing risks (Pintilie 2006). To compare cumulative incidence functions, we will use Gray's test (Gray 1988). To assess the effects of covariates on the cumulative incidence function, we will use the univariate and multicovariate proportional hazards models of Fine and Gray (1999). For the multivariate competing risk regression model analysis, we will first evaluate a model with all covariates of interest and then keep only those with p-values less than 0.10 in the final multicovariate model. In analyzing DFS, recurrence, metastasis or death will be considered as an event. Kaplan-Meier method will be used to estimate DFS time and OS time and log-rank test will be used to compare DFS time and OS time between or among patient demographic/clinic-pathological characteristics groups and treatment groups. Cox regression model will be employed to assess the effect of important covariates and treatment on DFS time and OS time.

9.8 Cytokine analysis, homocysteine levels and lipid profiles

The luminex assay will be performed for blood drawn pre- and post-radiation and the 79 cytokine profiles generated will be analyzed. The endpoint of this analysis will log base 2 of the ratio of the post-radiation cytokine level to the pre-radiation level. Differentially expressed cytokine levels will be investigated between the shorter- and longer-course radiation patients as well as between patients who suffer from lymphedema versus patients not suffering from lymphedema using a two-tailed pooled t-test for independent samples. Multiple testing will be handled using

Hochberg's step-up method. A Shapiro-Wilk test will objectively assess the normality of the data and these findings will be visually confirmed by inspecting normal probability Q-Q plots. If the data are not normally distributed, we may consider data transformation before using t-test or Wilcoxon rank sum test to investigate differences between two groups.

For each patient the ratio of the standardized sum of the Th1 cytokines to the standardized sum of the Th2 cytokines will be calculated pre- and post-RNI and the change from baseline determined. The change from baseline will be compared between the short- and standard RNI patients as well as between patients who suffer from lymphedema versus patients not suffering from lymphedema using a t-test or Wilcoxon sum rank test. Unsupervised hierarchical clustering using Euclidean distance and average linkage may be used to split the dataset of cytokines into subgroups. A heat-map may be generated to visualize and assist interpretation of the hierarchical clustering results.

As an exploratory objective, we may attempt to validate the only existing lymphedema model (Lin 2012) by fitting a logistic regression model within treatment group as well as treatment groups combined that includes the predictors: FGFb, IL4, IL10, TNFb, TGFb, and Leptin. Asymptotic 95% CIs for the area under the receiver operating characteristic curves (AUC) and the difference between the AUCs of the two treatment groups may be estimated using generalized U-statistics theory (DeLong 1988).

Homocysteine levels and lipid profiles will be measured from blood drawn prior to radiation. Homocysteine levels will be compared between patients who develop lymphedema and those who do not using two-sample t-test or Wilcoxon rank sum test whichever appropriate. We will also evaluate the effect of lipid profiles on time to locoregional recurrence using the univariate and multi-covariate proportional hazards models of Fine and Gray and examine the association between lipid profiles and acute toxicity. We hypothesize that high HDL and low LDL are each associated with reduced acute toxicity measures.

Analysis of cytokines, homocysteine levels and lipid profiles will occur after N= 250 patients had been enrolled on the trial. This is not applicable for the Cancer Network Site patients or Orlando Health.

9.9 Echocardiography with global longitudinal strain measurements.

With 30 patients in each group receiving radiation on the left side who will have echocardiography with global longitudinal strain measurements, we will be able to

estimate the change of the cardiac strain at each follow-up time from pre-treatment measures with a two-sided 95.0% confidence interval extending 0.895% from the observed mean, assuming that the standard deviation is 2.5%. On the other hand, if we only have 15 patients in each group receiving radiation on the left side with echocardiography with global longitudinal strain measurements, we will be able to estimate the change of the strain with a two-sided 95.0% confidence interval extending 1.265% from the observed mean, assuming that the standard deviation is 2.5%.Due to the longitudinal nature of the measures of various cardiac strain markers by image and by blood, repeated measures analysis may be employed to assess the effect of treatment over time on these measures, adjusted for other important covariates.

9.10 Analysis of H2AX data

As described in Dr. Stauder's IRG grant, the analysis of the g-H2AX data will be descriptive plots showing the foci per cell measured in up to 3 timepoints per patient, displayed in separate "spaghetti" plots. The anticipated pattern is an increase in the number of foci between sample 1 (baseline) and sample 2 (end RT), and a decrease in foci per cell in sample 3 (3-month follow-up) compared to sample 2 but greater than the number of foci seen in sample 1. Differences in the relative fluorescence in the PBL between patient groups will be compared using a McNemar's paired test of binomial proportions, specifically, the γ-H2AX retention in PBL at each collection time point. The difference in the number of foci between collection points will be analyzed by McNemar's paired binomial proportion test. This method does not rely on normality of data distribution. It incorporates within patient intra-correlation. Using this method for a 2-sided test with type I error level at 0.05, a sample size of 70 patients will have power of 80.3% to detect the 15% percentage difference in the relative fluorescence in the PBL, from 10% at baseline to 25% at week 1, with an intra-correlation of 0.4. For example, a difference of the relative fluorescence of 20% or greater, a sample size of 50 patients will have a power of 86.3 (see Table).

Sample 1	Sample 2	N Pairs	Power at correlation of			
			0.2	0.3	0.4	0.5
10%	25%	50	0.481	0.535	0.605	0.694
10%	25%	60	0.585	0.643	0.718	0.814
10%	25%	70	0.672	0.732	0.803	0.889
10%	25%	80	0.741	0.802	0.865	0.935
10%	25%	90	0.796	0.854	0.91	0.963
10%	25%	100	0.841	0.892	0.94	0.979
10%	30%	50	0.728	0.789	0.863	0.944
10%	30%	60	0.824	0.875	0.931	0.984
10%	30%	70	0.887	0.929	0.967	0.996
10%	30%	80	0.928	0.96	0.984	0.999
10%	30%	90	0.954	0.978	0.993	>.999
10%	30%	100	0.972	0.988	0.997	>.999

Analysis of H2AX data will occur after N= 250 patients have been enrolled on the trial. This is not applicable for the Cancer Network Site patients or Orlando Health.

9.11 Analysis for Clonal Hematopoiesis (CH and adverse treatment toxicity and outcomes

We will test whether a greater proportion of individuals harbor detectable CH mutations following RT as compared to before RT. Secondary outcomes investigated will focus on understanding the genetic architecture of CH mutations before and after RT by examining the number of CH mutations per individual, type of mutation (e.g. nonsense, indel, splice-site, missense), involved gene type (e.g. DNA damage repair), and distribution of involved genes (e.g. proportion of DNMT3A mutations). Additionally, we will examine the evolution of CH mutations over time as measured by the frequency and burden (measured by alternate allele fraction) of mutations using serial samples within individuals. We will utilize a paired sample analysis (i.e. generalized estimating equations). We will also test whether RT confers an increased risk of acute or chronic grade ≥2 and ≥3 toxicities, specifically cardiac and pulmonary toxicity, as well as cardiac biomarkers and function (determined by echocardiography). We will secondarily examine risk of hematologic cancer and acute or chronic grade ≥2 and ≥3 hematologic toxicity (Common Terminology Criteria for Adverse Events (CTCAE) v5). Analyses will be conducted

using multi-variable adjusted logistic regression techniques. Other secondary outcomes will include other acute or chronic grade ≥2 and ≥3 toxicity and oncologic outcomes, including recurrence, time to recurrence, overall survival and cancer-specific survival. Secondary exposures include induced CH mutations only and any CH mutations post-treatment. Analyses will be adjusted for potential confounding factors, such as age, gender, prior radiation exposure, chemotherapy, and prior tobacco use.

9.12 Additional analysis

Other methods of analysis, as appropriate, may be employed to analyze the data collected in this study, such as upper limb functions measured by QuickDASH-9 and patient quality of life (QOL) using EQ-5D and PROMIS Fatigue, and reconstructive complications separately in patient with neoadjuvant RNI and patients with adjuvant RNI. In general, these outcomes will be summarized at the end of the study using frequency tabulation for categorical variables or descriptive statistics (mean, standard deviation, median, and range) for continuous variables. Comparisons of these variables and any change of these variables between two time points between treatment groups may be made by chi-squared test/Fisher's exact test or Wilcoxon rank sum test, when appropriate. These functions and QOL will be measured repeatedly over time therefore repeated measures analysis may be employed to assess the effect of treatment over time on these measures. Statistical significance will be defined as $p < 0.05$ for all analyses.

9.13 Exploratory Analyses

Banked blood samples from 20 patients will be examined for feasibility of detecting radiation generated immune proteins to generate exploratory data irradiation for hypothesis generation and sample size estimates. No comparisons to study endpoints will be performed without further amendment.

Pathologic banked lymph node specimens from 5-10 patients who received pre-operative radiation and were surgical staged ypN1, ypN1mic or ypN0 itc+ and 5-10 nodal specimens (pN1, pN1mic or pN0 itc+) from patients who received post-operative radiation will be examined using multi-plexed immunohistochemistry to test feasibility of the technology on nodal samples and generate exploratory data regarding the effect of nodal irradiation for hypothesis generation and

sample size estimates. Analysis will be limited to patients who either consented on the front door consent to permit analysis of their tissue or who agreed to allow us to evaluate their lymph nodes as an optional procedure for this protocol. No comparisons to study endpoints will be performed without further amendment.

Pathologic banked tumor specimens from up to 100 patients who agreed to optional lipid studies on this protocol will be stained for correlative proteins involved in lipid regulation. Up to 10 unstained slides, per participant, will be assessed. Slides labelled with study ID will be shipped to Dr. Erik Nelson at University of Illinois for staining after MTA is in place. The MD Anderson participant study ID number (OnCore sequence number) does not contain any participant identifiable information and is instead used as a mechanism to crosslink participant data. Results will be correlated to tumor and patient variables [2.2 primary objective and 2.3 and secondary objectives]. No comparisons to the primary study endpoint [2.1] will be made without further amendment. Specific stains include: 1) CYP27A1, 2) CYP7B1, 3) FXR, 4) LXR (alpha and beta), 5) Small Heterodimer Partner (SHP), 6) LRH1, 7) TLX, 8) ABCA1, 9) ABCG1, 10) CD68 (macrophage), 11) CD11B (myeloid cell), 12) CD66 (neutrophil), 13) CD4, 14) FOXP3, 15) CD8, 16) GZMB.

9.14 Data Confidentiality Plan

All laboratory and clinical data gathered in this protocol will be stored in a password-protected database. Access to the database is only available to individuals directly involved in the study.

9.15 DSMB Monitoring Plan

The statistical team will generate posterior probabilities and predictive probabilities for monitoring time-to-lymphedema as well as posterior probabilities for toxicity every six months during the study and provide the information to DSMB for discussion.

10 Data Management:

This study is a collective effort of multiple centers including MD Anderson Cancer Center and Houston- Area Locations, Cancer Network sites, and Orlando Health. Cancer Network site staff will conduct the study in accordance with institutional and Cancer Network Research trial guidelines. Orlando Health site staff will follow the Data Management Plan (DQMP).

11 REFERENCES

1. Ancukiewicz, M., T. A. Russell, J. Otoole, M. Specht, M. Singer, A. Kelada, C. D. Murphy, J. Pogachar, V. Gioioso, M. Patel, M. Skolny, B. L. Smith and A. G. Taghian (2011). "Standardized method for quantification of developing lymphedema in patients treated for breast cancer." Int J Radiat Oncol Biol Phys **79**(5): 1436-1443.
2. Appelt, A.L., I.R. Vogelius IR, S.M. Bentzen (2013). “Modern hypofractionation schedules for tangential whole breast irradiation decrease the fraction size-corrected dose to the heart.” Clin Oncol **25**(3):147–52
3. Baumann DP, Crosby MA, Selber JC, Garvey PB, Sacks JM, Adelman DM, Villa MT, Feng L, Robb GL (2011). Optimal timing of delayed free lower abdominal flap breast reconstruction after postmastectomy radiation therapy. Plast Reconstr Surg **127**(3):1100-6.
4. Brooks, R. (1996). "EuroQol: the current state of play." Health Policy **37**(1): 53-72.
5. Cella D, Riley W, Stone A, Rothrock N, Reeve B, Yount S, Amtmann D, Bode R, Buysse D, Choi S, Cook K, Devellis R, DeWalt D, Fries JF, Gershon R, Hahn EA, Lai JS, Pilkonis P, Revicki D, Rose M, Weinfurt K, Hays R and the PROMIS Cooperative Group (2010). “The Patient-Reported Outcomes Measurement Information System (PROMIS) developed and tested its first wave of adult self-reported health outcome item banks: 2005-2008.” Journal of Clinical Epidemiology 63(11), 1179-1194.
6. Chua, M. L. K., N. Somaiah, R. A’Hern, S. Davies, L. Gothard, J. Yarnold and K. Rothkamm (2011). "Residual DNA and chromosomal damage in ex vivo irradiated blood lymphocytes correlated with late normal tissue response to breast radiotherapy." Radiotherapy and Oncology 99(3): 362-366.

7. Coster, S., K. Poole and L. J. Fallowfield (2001). "The validation of a quality of life scale to assess the impact of arm morbidity in breast cancer patients post-operatively." Breast Cancer Res Treat 68(3): 273-282.

8. D'Agostino, R. B., R. S. Vasan, M. J. Pencina, et al. (2008) “General cardiovascular risk profile for use in primary care: the Framingham Heart Study.” Circulation 117(6):743-753.

9. DeLong, E. R., D. M. DeLong and D. L. Clarke-Pearson (1988). "Comparing the areas under two or more correlated receiver operating characteristic curves: a nonparametric approach." Biometrics 44(3): 837-845.

10. Deltombe, T., J. Jamart, S. Recloux, C. Legrand, N. Vandenbroeck, S. Theys and P. Hanson (2007). "Reliability and limits of agreement of circumferential, water displacement, and optoelectronic volumetry in the measurement of upper limb lymphedema." Lymphology **40**(1): 26-34.

11. EBCTCG (Early Breast Cancer Trialists Collaborative Group), P. McGale, C. Taylor, C. Correa, D. Cutter, F. Duane, M. Ewertz, R. Gray, G. Mannu, R. Peto, T. Whelan, Y. Wang, Z. Wang and S. Darby (2014). "Effect of radiotherapy after mastectomy and axillary surgery on 10-year recurrence and 20-year breast cancer mortality: meta-analysis of individual patient data for 8135 women in 22 randomised trials." Lancet **383**(9935): 2127-2135.

12. Erickson, V. S., M. L. Pearson, P. A. Ganz, J. Adams and K. L. Kahn (2001). "Arm edema in breast cancer patients." J Natl Cancer Inst **93**(2): 96-111.

13. EuroQol, G. (1990). "EuroQol--a new facility for the measurement of health-related quality of life." Health Policy **16**(3): 199-208.

14. Fine J.P. and R. J. Gray (1999). A proportional hazards model for the subdistribution of a competing risk.” J Am Stat Assoc **94**:496–509.

15. Finkelstein, S. E., R. Timmerman, W. H. McBride, D. Schaue, S. E. Hoffe, C. A. Mantz and G. D. Wilson (2011). "The confluence of stereotactic ablative radiotherapy and tumor immunology." Clin Dev Immunol **2011**: 439752.

16. Gabel, C. P., M. Yelland, M. Melloh and B. Burkett (2009). "A modified QuickDASH-9 provides a valid outcome instrument for upper limb function." BMC Musculoskelet Disord **10**: 161.

17. Garcia SF, Cella D, Clauser SB, Flynn KE, Lad T, Lai JS, Reeve BB, Smith AW, Stone AA, Weinfurt K. (2007). “Standardizing patient-reported outcomes assessment in cancer clinical trials: a

patientreported outcomes measurement information system initiative." Journal of Clinical Oncology 25(32), 5106-5112.

18. Garcia-Tevijano, E. R., C. Berasain, J. A. Rodriguez, F. J. Corrales, R. Arias R, A. Martin-Duce A (2001) Hypertension **38**(5):1217-21.
19. Giacalone PL, Rathat G, Daures JP, Benos P, Azria D, Rouleau C (2010). New concept for immediate breast reconstruction for invasive cancers: feasibility, oncological safety and esthetic outcome of post-neoadjuvant therapy immediate breast reconstruction versus delayed breast reconstruction: a prospective pilot study. Breast Cancer Res Treat **122**(2):439-51.
20. Gough, M. J., M. R. Crittenden, M. Sarff, P. Pang, S. K. Seung, J. T. Vetto, H. M. Hu, W. L. Redmond, J. Holland and A. D. Weinberg (2010). "Adjuvant therapy with agonistic antibodies to CD134 (OX40) increases local control after surgical or radiation therapy of cancer in mice." J Immunother **33**(8): 798-809.
21. Gray, R.J. (1988). "A class of K-sample tests for comparing the cumulative incidence of a competing risk." Ann Stat **3**:1141–1154.
22. Haviland, J. S., J. R. Owen, J. A. Dewar, R. K. Agrawal, J. Barrett, P. J. Barrett-Lee, H. J. Dobbs, P. Hopwood, P. A. Lawton, B. J. Magee, J. Mills, S. Simmons, M. A. Sydenham, K. Venables, J. M. Bliss, J. R. Yarnold and S. T. Group (2013). "The UK Standardisation of Breast Radiotherapy (START) trials of radiotherapy hypofractionation for treatment of early breast cancer: 10-year follow-up results of two randomised controlled trials." Lancet Oncol **14**(11): 1086-1094.
23. Ho AL, Tyldesley S, Macadam SA, Lennox PA (2012). Skin-sparing mastectomy and immediate autologous breast reconstruction in locally advanced breast cancer patients: a UBC perspective. Ann Surg Oncol **19**(3):892-900.
24. Jagsi, R., P. Abrahamse, M. Morrow, J. J. Griggs, K. Schwartz and S. J. Katz (2009). "Postmastectomy radiotherapy for breast cancer: patterns, correlates, communication, and insights into the decision process." Cancer **115**(6): 1185-1193.
25. Jagsi, R., J. A. Pottow, K. A. Griffith, C. Bradley, A. S. Hamilton, J. Graff, S. J. Katz and S. T. Hawley (2014). "Long-term financial burden of breast cancer: experiences of a diverse cohort of survivors identified through population-based registries." J Clin Oncol **32**(12): 1269-1276.
26. Jagsi R, Li Y, Morrow M, Janz N, Alderman A, Graff J, Hamilton A, Katz S, and Hawley S (2015). "Patient-reported quality of life and satisfaction with cosmetic outcomes after breast conservation

and mastectomy with and without reconstruction: results of a survey of breast cancer survivors." Ann Surg **26**1(6):1198-206.

27. Jagsi R (2019). "Hypofractionated Radiotherapy After Mastectomy: A New Frontier?" Lancet Oncol **20**(3)313-315.
28. Marta, G. N., and P. Poortmans. (2019). "Moderately hypofractionated Breast Raidation Therapy: Is More Evidence Needed?" Lancet Oncol **20**(5)e226.
29. Busque L, Mio R, Mattioli J, et al. Nonrandom X-inactivation patterns in normal females: lyonization ratios vary with age. *Blood.* 1996;88(1):59-65.
30. Jacobs KB, Yeager M, Zhou W, et al. Detectable clonal mosaicism and its relationship to aging and cancer. *Nat Genet.* 2012;44(6):651-658.
31. Buscarlet M, Provost S, Zada YF, et al. DNMT3A and TET2 dominate clonal hematopoiesis and demonstrate benign phenotypes and different genetic predispositions. *Blood.* 2017;130(6):753-762.
32. Jaiswal S, Fontanillas P, Flannick J, et al. Age-related clonal hematopoiesis associated with adverse outcomes. *N Engl J Med.* 2014;371(26):2488-2498.
33. Fuster JJ, MacLauchlan S, Zuriaga MA, et al. Clonal hematopoiesis associated with TET2 deficiency accelerates atherosclerosis development in mice. *Science.* 2017;355(6327):842-847.
34. Coombs CC, Zehir A, Devlin SM, et al. Therapy-Related Clonal Hematopoiesis in Patients with Non-hematologic Cancers Is Common and Associated with Adverse Clinical Outcomes. *Cell Stem Cell.* 2017;21(3):374-382 e374.
35. Takahashi K, Wang F, Kantarjian H, et al. Preleukaemic clonal haemopoiesis and risk of therapy-related myeloid neoplasms: a case-control study. *Lancet Oncol.* 2017;18(1):100-111.
36. Kronowitz SJ, Lam C, Tereffe W, et al. Plast Reconstr Surg. 2011 Jun;127(6):2154-66. doi: 10.1097/PRS.0b013e3182131b8e
37. Lacerda, L. J., P. Reddy, D. Liu, R. Larson, L. Li, H. Masuda, T. Brewer, B.G. Debeb, W. Xu, G. N. Hortobagyi, T. A. Buchholz, N. T. Ueno, W. A. Woodward (2014). "Simvastatin radiosensitizes differentiated and stem-like breast cancer cel lines and is associated with imprved local control in inflammmatory breast cancer patients treated with postmastectomy radidation." Stem Cells Trans Med **3** (7): 849-56.

38. Lee, Y., S. L. Auh, Y. Wang, B. Burnette, Y. Wang, Y. Meng, M. Beckett, R. Sharma, R. Chin, T. Tu, R. R. Weichselbaum and Y. X. Fu (2009). "Therapeutic effects of ablative radiation on local tumor require CD8+ T cells: changing strategies for cancer treatment." Blood **114**(3): 589-595.
39. Lin, S., J. Kim, M. J. Lee, L. Roche, N. L. Yang, P. S. Tsao and S. G. Rockson (2012). "Prospective transcriptomic pathway analysis of human lymphatic vascular insufficiency: identification and validation of a circulating biomarker panel." PLoS One **7**(12): e52021.
40. Markman, M. and R. Luce (2010). "Impact of the cost of cancer treatment: an internet-based survey." J Oncol Pract **6**(2): 69-73.
41. McCully, K.S (2005). "Hyperhomocysteinemia and arteriosclerosis: historical perspectives." Clin Chem Lab Med **43**(10):980-6.
42. McWayne, J. and S. P. Heiney (2005). "Psychologic and social sequelae of secondary lymphedema: a review." Cancer **104**(3): 457-466.
43. Monrigal E, Dauplat J, Gimbergues P, Le Bouedec G, Peyronie M, Achard JL, Chollet P, Mouret-Reynier MA, Nabholtz JM, Pomel C (2011). Mastectomy with immediate breast reconstruction after neoadjuvant chemotherapy and radiation therapy. A new option for patients with operable invasive breast cancer. Results of a 20 years single institution study. Eur J Surg Oncol **37**(10):864-70.
44. Nestle-Krämling C, Bölke E, Budach W, Andree C (2016). Breast reconstruction after neoadjuvant radio chemotherapy: review and personal technique IDEAL concept REV-EJMR-D-15-00268. Eur J Med Res;**21**(1):24.
45. Otte M, Nestle-Krämling C, Fertsch S, Hagouan M, Munder B, Richrath P, Stambera P, Abu-Ghazaleh A, Andree C (2016). Conservative mastectomies and Immediate-DElayed AutoLogous (IDEAL) breast reconstruction: the DIEP flap. Gland Surg **5**(1):24-31.
46. Paillocher N, Florczak AS, Richard M, Classe JM, Oger AS, Raro P, Wernert R, Lorimier G (2016). Evaluation of mastectomy with immediate autologous latissimus dorsi breast reconstruction following neoadjuvant chemotherapy and radiation therapy: A single institution study of 111 cases of invasive breast carcinoma. Eur J Surg Oncol **42**(7):949-55.
47. Pickard AS, Wilke CT, Lin HW, Lloyd A (2007). "Health utilities using the EQ-5D in studies of cancer." Pharmacoeconomics. **25**(5):365-384.
48. Pintile, M (2006). Competing risks: a practical perspective. Wiley, Hoboken.

49. Reilly, M. C., A. S. Zbrozek and E. M. Dukes (1993). "The validity and reproducibility of a work productivity and activity impairment instrument." Pharmacoeconomics **4**(5): 353-365.
50. Reits, E. A., J. W. Hodge, C. A. Herberts, T. A. Groothuis, M. Chakraborty, E. K. Wansley, K. Camphausen, R. M. Luiten, A. H. de Ru, J. Neijssen, A. Griekspoor, E. Mesman, F. A. Verreck, H. Spits, J. Schlom, P. van Veelen and J. J. Neefjes (2006). "Radiation modulates the peptide repertoire, enhances MHC class I expression, and induces successful antitumor immunotherapy." J Exp Med **203**(5): 1259-1271.
51. Riet FG, Fayard F, Arriagada R, Santos MA, Bourgier C, Ferchiou M, Heymann S, Delaloge S, Mazouni C, Dunant A, Rivera S (2017). Preoperative radiotherapy in breast cancer patients: 32 years of follow-up. Eur J Cancer **76**:45-51.
52. Shah, C., D. Arthur, J. Riutta, P. Whitworth and F. A. Vicini (2012). "Breast-cancer related lymphedema: a review of procedure-specific incidence rates, clinical assessment AIDS, treatment paradigms, and risk reduction." Breast J **18**(4): 357-361.
53. Shaitelman, S. F., P. J. Schlembach, I. Arzu, M. Ballo, E. S., Bloom, D. Buchholz, G. M. Chronowski, T. Dvorak, E. Grade, K. E. Hoffman, P. Kelly, M. Ludwig, G. H. Perkins, V. Reed, S. Shah, M. C. Stauder, E. A. Strom, W. Tereffe, W. A. Woodward, J. Ensor, D. Baumann, A. M. Thokmpson, D. Amaya, T. Davis, W. Guerra, L. Hamblin, G. Hortobagy, K.K. Hunt, T.A. Buchholz and B. Smith "Acute and Short-term Toxic effects of Conventionally Fractionated vs hypofractionated Whole-Breast Irrradiation: A Randomized Clinical Trial." JAMA Oncol **1**(7): 931-941.
54. Shirvani, S. M., I. W. Pan, T. A. Buchholz, Y. C. Shih, K. E. Hoffman, S. H. Giordano and B. D. Smith (2011). "Impact of evidence-based clinical guidelines on the adoption of postmastectomy radiation in older women." Cancer **117**(20): 4595-4605.
55. Smith, B. D., S. M. Bentzen, C. R. Correa, C. A. Hahn, P. H. Hardenbergh, G. S. Ibbott, B. McCormick, J. R. McQueen, L. J. Pierce, S. N. Powell, A. Recht, A. G. Taghian, F. A. Vicini, J. R. White and B. G. Haffty (2011). "Fractionation for whole breast irradiation: an American Society for Radiation Oncology (ASTRO) evidence-based guideline." Int J Radiat Oncol Biol Phys **81**(1): 59-68.
56. Smith, B. D., J. R. Bellon, R. Blitzblau, G. Freedman, B. Haffty, C. Hahn, F. Halberg, K. Hoffman, K. Horst, J.Moran, C. Patton , J. Perlmutter, L. Warren, T. Whelan, J.L. Wright , R. Jagsi R. (2018). "Radiation therapy for the whole breast: Executive summary of an American Society for Radiation Oncology (ASTRO) evidence-based guideline." Pract Radiat Oncol **8**(3):145-152.

57. Smith GL, Volk RJ, Lowenstein LM, Peterson SK, Rieber AG, Checka C, CHristopherson KM, Jagsi R, Giordano SH, Mendoza TR (2019). “ENRICH: Validating a multidimensional patient-reported financial toxicity measure.” J Clin Oncol **37**(27):s153.
58. Teo I, Reece GP, Christie IC, Guindani M, Markey MK, Heinberg LJ, Crosby MA, Fingeret MC (2016). Body image and quality of life of breast cancer patients: influence of timing and stage of breast reconstruction. Psychooncology **25**(9):1106-12.
59. Thiruchelvam P, Hadjiminas D, Cleator S, Wood S, Jallali N, Kirby A, James S, Leff D, MacNeill F, on behalf of the PRADA trial working group (2017). Primary Radiotherapy And DIEP flAp reconstruction. The PRADA study. Abstract; American Society of Breast Surgeons Meeting.
60. van Oorschot, B., S. E. Hovingh, P. D. Moerland, J. P. Medema, L. J. A. Stalpers, H. Vrieling and N. A. P. Franken (2014) "Reduced Activity of Double-Strand Break Repair Genes in Prostate Cancer Patients With Late Normal Tissue Radiation Toxicity." International Journal of Radiation Oncology • Biology • Physics 88(3): 664-670
61. Van Parijs, H., G. Miedema, V. Vinh-Hung, S. Verbanck, N. Adriaenssens, D. Kerkhove, T. Reynders, D. Schuermans, K. Leysen, S. Hanon, G. Van Camp, W. Vincken, G. Storme, D. Verellen and M. De Ridder (2012). "Short course radiotherapy with simultaneous integrated boost for stage I-II breast cancer, early toxicities of a randomized clinical trial." Radiat Oncol **7**: 80.
62. Wang, S-L., H. Fang, Y. Sang, W. Wang, C. Hu, Y. Liu, J. Jin, X. Liu, Z. Yu, H. Ren, N. Li, N. Lu, Yu. Tang, Y. Tang, S. Qi, G. Sun, R. Peng, S. Li, B. Chen, Y. Yang, Y. Li (2019). “Hypofractionated versus conventional fractionated postmastectomy rdiotherapy for patients with high-risk breast cancer: a randomised non-inferiority, open-label phase 3 trial.” Lancet Oncol **20**(3):352-60.
63. Warren, L. E., C. L. Miller, N. Horick, M. N. Skolny, L. S. Jammallo, B. T. Sadek, M. N. Shenouda, J. A. O'Toole, S. M. MacDonald, M. C. Specht and A. G. Taghian (2014). "The impact of radiation therapy on the risk of lymphedema after treatment for breast cancer: a prospective cohort study." Int J Radiat Oncol Biol Phys **88**(3): 565-571.
64. Wetzig, N., P. G. Gill, D. Zannino, M. R. Stockler, V. Gebski, O. Ung, I. Campbell and R. J. Simes (2014). "Sentinel Lymph Node Based Management or Routine Axillary Clearance? Three-year Outcomes of the RACS Sentinel Node Biopsy Versus Axillary Clearance (SNAC) 1 Trial." Ann Surg Oncol. E-pub 2014 Oct 15.

65. Whelan, T. J., J. P. Pignol, M. N. Levine, J. A. Julian, R. MacKenzie, S. Parpia, W. Shelley, L. Grimard, J. Bowen, H. Lukka, F. Perera, A. Fyles, K. Schneider, S. Gulavita and C. Freeman (2010). "Long-term results of hypofractionated radiation therapy for breast cancer." N Engl J Med **362**(6): 513-520.
66. Whelan, T.J., I. Ackerman, J.W. Chapman, B. Chua, A.. Nabid, K.A. Vallis, J.R., White, P. Rousseau, A. Fortin, L.J. Pierce, L. Manchul, P. Craighead, M.C. Nolan, J. Bowen, D.R. McCready, Kk.I. Pritchard, M.N. Leine, W. Parulekar. "NCIC-CTG MA.20: an intergroup trial of regional nodal irradiation in early breast cancer." J Clin Onc ASCO Annual Meeting Proceedings 2011:29.
67. Wolfe, A. R. R. L. Atkinson, J. P. Reddy, b. G. Debeb, R. Larson, L. Li, H. Masuda, T. Brewer,B. J. Atkinson, A. Brewster, N. T. Uneo, W. A. Woodward (2015) "High-density and very-low-density lipoprotein have opposing roles in regulating tumor-initiating cells and sensitivity to radiation in inflammatory breast cancer" Int J Radiat Oncol Biol Phys **91** (5): 1072-80.
68. Woodward, W. A., J. B. Durand, S. L. Tucker, E. A. Strom, G. H. Perkins, J. Oh, L. Arriaga, D. Domain and T. A. Buchholz (2008). "Prospective analysis of carotid artery flow in breast cancer patients treated with supraclavicular irradiation 8 or more years previously: no increase in ipsilateral carotid stenosis after radiation noted." Cancer **112**(2): 268-273.
69. Zinzindohoué C, Bertrand P, Michel A, Monrigal E, Miramand B, Sterckers N, Faure C, Charitansky H, Gutowski M, Cohen M, Houvenaeghel G, Trentini F, Raro P, Daures JP, Lacombe S (2016). A Prospective Study on Skin-Sparing Mastectomy for Immediate Breast Reconstruction with Latissimus Dorsi Flap After Neoadjuvant Chemotherapy and Radiotherapy in Invasive Breast Carcinoma. Ann Surg Oncol **23**(7):2350-6.

Protocol Documents

Informed Consent

Perometer measurement of Arm Volume

EQ-5D-3L (English + Spanish versions)

QuickDASH-9

PROMIS Fatigue Short form 6a

Satisfaction with cosmetic outcomes questions

Patient-reported arm function questions (Arm Function Fact B+4)

QuickDASH-9 – Spanish version

PROMIS Fatigue Short form 6a – Spanish version

Satisfaction with cosmetic outcomes questions – Spanish version

Informational flyer for patients (applicable only for Lymphedema Cohort)

Informational video for patients (applicable only for Lymphedema Cohort)

Economic Strain and Resilience in Cancer- Financial Well- Being instrument

Patient- reported arm function questions (Arm Function Fact B+4) – Spanish

Dosimetry Form

Follow-up and Adverse Events

Data Quality Management Plan
