# Supplementary material for: Premastectomy Radiotherapy and Immediate Breast Reconstruction: A Randomized Clinical Trial
Source: JAMA Netw Open. 2024 Apr 5;7(4):e245217. doi: 10.1001/jamanetworkopen.2024.5217 (PMC10998161; doi:10.1001/jamanetworkopen.2024.5217)
Supplement: Supplement 2. — Data Sharing Statement [file jamanetwopen-e245217-s002.pdf]

Data Sharing Statement

Schaverien. Premastectomy Radiotherapy and Immediate Breast Reconstruction. *JAMA Netw Open*. Published April 05, 2024. doi:10.1001/jamanetworkopen.2024.5217

Data
Data available: No
